# Sexual and reproductive health information and referrals for resettled refugee women: A survey of resettlement agencies in the United States

**DOI:** 10.1371/journal.pmed.1003579

**Published:** 2021-05-03

**Authors:** Tonya Katcher, Rebecca Thimmesch, Alison Spitz, Leena Kulkarni, Neelima Panth, Arlen Weiner, Michelle Woodford Martin

**Affiliations:** 1 Advocates for Youth, Washington, District of Columbia, United States of America; 2 International Rescue Committee, Atlanta Office, Atlanta, Georgia, United States of America; 3 Harvard TH Chan School of Public Health, Boston, Massachussetts, United States of America; 4 Yale University School of Medicine, New Haven, Connecticut, United States of America; International Organization for Migration, SRI LANKA

## Abstract

**Background:**

Refugee resettlement offices are the first point of contact for newly arrived refugees and play a significant role in helping refugees acclimate and settle into life in the United States. Available literature suggests that refugee women are vulnerable to poor sexual and reproductive health (SRH) outcomes, including sexually transmitted infections and HIV infections as well as adverse pregnancy outcomes, but little is known about the role that refugee resettlement offices play in supporting refugee women’s SRH. This study examines the capacity and interest of resettlement offices in providing SRH information and referrals to newly arrived refugees.

**Methods and findings:**

The research team conducted an online survey of staff members at refugee resettlement offices throughout the US in 2018 to determine (1) available SRH resources and workshops; (2) referrals to and assistance with making appointments for SRH and primary care appointments; (3) barriers to addressing SRH needs of clients; and (4) interest in building the capacity of office staff to address SRH issues. The survey was created for this study and had not been previously used or validated. Survey data underwent descriptive analysis. A total of 236 resettlement offices were contacted, with responses from 100 offices, for a total response rate of 42%. Fifteen percent (*N* = 15) of refugee resettlement agencies (RRAs) who responded to the survey provide materials about SRH to clients, and 49% (*N* = 49) incorporate sexual health into the classes they provide to newly arrived refugee clients. Moreover, 12% (*N* = 12) of responding RRAs screen clients for pregnancy intention, and 20% (*N* = 20) directly refer to contraceptive care and services. This study is limited by the response rate of the survey; no conclusions can be drawn about those offices that did not respond. In addition, the survey instrument was not validated against any other sources of information about the practices of refugee resettlement offices.

**Conclusions:**

In this study, we observed that many resettlement offices do not routinely provide information or referrals for SRH needs. Responding offices cite lack of time and competing priorities as major barriers to providing SRH education and referrals to clients.

## Abstract 2

In a survey of US refugee resettlement offices, Tonya Katcher and colleagues investigate how resettlement offices support sexual and reproductive health of clients.

## Author summary

**Why was this study done?:**

- Women who are recently resettled refugees often come from settings in which there is limited access to sexual and reproductive health (SRH) care services.
- No research has been done to date regarding the extent to which refugee resettlement offices are screening women for reproductive health needs, providing SRH education, or referring to medical providers for SRH care.

**What did the researchers do and find?:**

- Researchers conducted a survey of all of the refugee resettlement agencies in the United States to assess their practices regarding screening, education, and referrals for SRH needs. The survey also assessed perceived barriers to providing SRH screening, education and referrals, and respondents interested in improving the capacity of their office to provide these services.
- The study found that while nearly half of responding offices provide some SRH-related education through classes or workshops to newly arrived refugees, few provide educational materials or patient referrals for care.
- Responding offices cited lack of time and competing priorities as major barriers to providing SRH information and referrals to clients.

**What do these findings mean?:**

- Recently resettled refugee women are generally not receiving SRH screening, education, and referrals from refugee resettlement offices.
- Resettlement offices could better serve the SRH needs of refugee women.

## Introduction

Conflict and crisis have dire consequences on women and girls’ sexual and reproductive health (SRH) and rights. Women and girls affected by conflict often have limited access to SRH care and are particularly vulnerable to unintended pregnancies, which may lead to high rates of unsafe abortion, maternal mortality, stillbirth, and perinatal mortality [1–3]. While migration to the United States may offer enhanced opportunities for access to SRH services, little is known about the extent of information and clinical services available for women through refugee resettlement agencies (RRAs). A study from 2004 showed that 25% of newly resettled refugee women were pregnant or had a reproductive health problem, and many had missed routine preventive care including breast and cervical cancer screenings [4]. Another study, published in Canada, calculated that 26% of newly resettled refugee women had unmet contraceptive need, meaning they were sexually active and not wanting to become pregnant but not using a contraceptive method [5]. These studies underscore that newly resettled refugee women have significant SRH needs. However, to the best of our knowledge, the role that RRAs play in supporting refugee women’s SRH needs has not been investigated.

Refugee resettlement is undertaken by a public–private partnership between the US government and nonprofit organizations. There are currently 9 organizations with a network of local affiliates across the country that have cooperative agreements with the government to provide resettlement services. The initial services—those provided in the first 3 months after arrival—include housing, food, job placement, and medical care, and are funded by the Department of State’s Reception and Placement Program [6]. At the time of this study, the researchers identified 236 individual resettlement offices throughout the US. These offices assist newly arrived refugees with housing, obtaining education and employment, accessing public services, and acclimating to life in the US.

To better understand how these agencies support the SRH needs of their clients, Advocates for Youth (AFY), a nonprofit organization that supports efforts to improve the SRH of adolescents and young adults, jointly conducted a survey with the International Rescue Committee (IRC), a nonprofit organization that responds to the world’s most severe humanitarian crises, helps people to survive and rebuild their lives, and is one of the 9 refugee resettlement agencies in the US. This effort is part of a broader AFY project to increase awareness of, access to, and use of contraception among refugee young adults. The findings from this survey may help lay the groundwork for strengthening the capacity of refugee resettlement agencies to integrate SRH services into programming and support refugees’ ability to plan for their futures by planning their pregnancies and families.

## Methods

This project was designed and conducted by AFY, with the assistance of students from Harvard’s T.H. Chan School of Public Health and staff from the United States’ national office and the Atlanta, Georgia, office of the IRC. The survey instrument addressed 4 key areas: (1) available SRH resources and workshops; (2) referrals to and assistance with making appointments for SRH and primary care appointments; (3) barriers to addressing SRH needs of clients; and (4) interest in building the capacity of office staff to address SRH issues. The survey consisted of 29 yes/no or multiple-choice questions; all survey data were qualitative. The survey was created expressly for this study and was not previously used or validated. The survey included an introduction to the study, explaining how the data were intended to be used. The survey may be reviewed in supplemental information, under the document title S1 Survey. The survey data were intended to undergo descriptive analysis, which was conducted by AFY. The data analysis plan was created prior to administration of the survey, and no changes to the plan were made during data analysis.

The survey was conducted in 2 stages, first with non-IRC resettlement offices and later with IRC offices. The survey was conducted in this manner due to a concurrent collaboration between AFY and the IRC; IRC management requested to reach out to their own affiliates themselves, rather than having AFY contact them via phone or email, as was the protocol for other resettlement agency offices. Using the website of the United Nations High Committee on Refugees (available at http://www.unhcr.org/en-us/us-resettlement-agencies.html), the research team identified 210 unduplicated non-IRC refugee resettlement offices. Survey participants from each of those offices were recruited through direct phone or email outreach, with the goal that the survey be completed by one staff member familiar with the full range of services available at each office. In May and June of 2018, all 210 non-IRC offices were contacted by email or phone, a maximum of 3 times. Once an office was known to have responded to the survey, no further contacts were attempted for that office. The survey was conducted using SurveyMonkey, an online platform.

For the second stage of survey implementation with IRC offices, AFY staff worked with IRC national and Atlanta staff to make minor adaptations to survey questions to ensure alignment with IRC terminology. IRC national leadership directly invited, by email, each of the 26 IRC offices to participate in the survey. The survey was again conducted using SurveyMonkey, across a 3-week period in July and August 2018, with a request that the survey be completed by one person familiar with the range of SRH resources available at each office.

For both non-IRC and IRC surveys, data analysis was conducted on the SurveyMonkey platform. The results presented in this manuscript represent aggregated responses from both non-IRC and IRC offices.

As this survey gathered information exclusively about the practices and resources available in the respondents’ places of employment, the study protocol did not meet the criteria of human participants’ research. Per the guidelines and decision-making tool available at https://grants.nih.gov/policy/humansubjects/hs-decision.htm, the study did not require institutional review, thus it was not submitted to any institutional review board. The survey included a consent statement explaining the purposes of the survey and the risks and benefits of participating so that each respondent could choose whether or not to participate; the organizations as a whole were not approached to request consent for participation.

This study was reported per the guidelines published in “Standards for Reporting Qualitative Research,” as published in the journal Academic Medicine in September of 2014 [7]. The checklist is included in the supplemental materials, titled S1 Checklist.

## Results

Across all of the 236 non-IRC and IRC offices invited to participate, the survey collected 100 responses, for a response rate of 42%. The response rate among the 210 non-IRC offices was 38%, while the response rate from the 26 IRC offices was 77%. Some respondents answered some but not all of the questions on the survey; for any given question, the response rate was between 80% and 96%. All 9 of the resettlement agencies were represented among respondents, with IRC and the US Conference of Catholic Bishops affiliates accounting for the largest percentage of respondents (Table 1). Survey respondents included resettlement managers, coordinators and directors, executive directors, deputy directors or vice presidents, program managers, and case managers. Seventy-one percent of respondents provided the name of their office and contact information. Among those that did provide contact information, there were no known instances in which 2 people from the same office responded to the survey.

**Table 1:** Respondent characteristics.

*Organizational affiliation*: *(some respondents reported more than one organizational affiliation)*	Number (*n* = 100)	Percent
Church World Service	11	11%
Ethiopian Community Development Council	6	6%
Episcopal Migration Ministries	7	7%
Hebrew Immigrant Aid Society	12	12%
International Rescue Committee	20	20%
Lutheran Immigration and Refugee Service	9	9%
US Committee for Refugees and Immigrants	10	10%
US Conference of Catholic Bishops/Migration and Refugee Services	24	24%
World Relief	9	9%
*Professional role*:	Number (*n* = 100)	
Executive Director	18	18%
Vice President or Deputy Director	5	5%
Resettlement coordinator/manager/director	44	44%
Case manager	6	6%
Program manager	9	9%
Other	14	14%
No response	5	5%

### Available SRH resources or workshops

All survey respondents were asked whether their offices offered written materials (such as pamphlets or handouts) on SRH for clients (Table 2). Of the 95 offices who responded to this question, 15% (14) stated that their offices do have written materials, such as pamphlets or flyers. Of those offices that do have written SRH materials for clients, 92% (11) reported providing materials in at least one language other than English. Only 5% (5) of offices display any posters or signs that provide SRH information. Pamphlets and signs contained information on family planning/birth control and how to access clinical reproductive health services.

**Table 2:** Availability of SRH resources and education.

	Yes % (*n*)	No % (*n*)
Agency offers written materials with SRH information	15% (14)	85% (80)
Agency displays posters or signs that provide SRH information	5% (5)	95% (86)
Agency offers workshops that cover SRH topics	41% (37)	59% (54)

Forty-one percent (37) of offices offer workshops that cover SRH topics. Among those offices, 51% (18) offer workshops in a single session, and 11% (4) offer a multi-workshop SRH series. Forty-nine percent (17) also include SRH content in cultural orientation courses. Forty percent (14) of offices reported conducting classes at least 4 to 6 times per year. The most common topics covered include: family planning/birth control; the reproductive health system and how pregnancy occurs; and how to access clinical reproductive health services. Sixty-nine percent (24) of workshops are always or usually separated by gender, and 82% (28) are voluntary for clients.

Only 17% (15) of 86 responding offices have a health educator on staff. Only 12% (10) of agencies surveyed always or usually screen female clients upon intake for whether they want to become pregnant in the next year.

Among the offices that responded, 52% (44) reported developing partnerships with other organizations to provide SRH workshops. These other organizations included medical clinics and providers and local departments of health.

### Referrals for SRH and primary care

Almost all offices (90%, *N* = 76) reported being aware of nearby clinics where clients can get SRH services, but only 20% (17) reported that they refer clients to a clinic specifically for reproductive health services, including contraception and preconception care. However, when asked if the offices provided any additional assistance to clients who wished to obtain reproductive health services, 74% (59) of respondents reported providing assistance with making appointments; transportation to clinics; assistance obtaining medications; and assistance obtaining health insurance. Forty-four percent (35) of offices reported assisting their clients with having interpretation services during a clinical visit.

### Barriers to addressing SRH needs

Survey respondents were asked, “In your opinion, what are the barriers your office faces in ensuring women clients receive SRH information and services?” Primary responses included lack of time/too many competing priorities; lack of culturally and linguistically appropriate SRH materials; clients’ cultural backgrounds, attitudes, and beliefs about SRH; and clients’ lack of knowledge about reproductive health. Respondents cited materials and staff trainings as the most-needed resources in order to build their agencies’ capacities to promote sexual health services for clients.

### Interest in building office capacity

Seventy-three percent (60) of respondents reported either “yes” or “maybe” when asked if they thought their office would be interested in increasing its capacity to provide SRH information and referrals to clients (Table 3). Those who responded positively were asked to select which listed trainings would be of potential interest to them; respondents could choose all that applied. Thirty-six percent (27) selected general staff training about SRH, 19% (14) selected training specifically about birth control, and 64% (47) selected training about how to talk to clients about SRH. Additionally, 87% (64) of respondents stated offices needed written SRH materials (handouts, pamphlets) for clients.

**Table 3:** Interest in expanding agency capacity.

Would the agency be interested in expanding its capacity to provide SRH information and referrals to clients?	
Yes	32% (26)
Maybe	41% (34)
No	17% (14)
Respondent does not know	10% (8)

## Discussion

Refugee resettlement offices are typically refugees’ first points of contact with the social services and supports that are available to them upon arrival in the US. They assist newly arrived refugees with a number of primary needs, including housing, food assistance, cultural orientation, healthcare, and employment—needs which are clearly important to refugees’ ability to establish a new life in this country and to become self-supporting. This study examines the extent to which resettlement offices are addressing the SRH needs of newly arrived refugees, which could be accomplished by a nonmedically trained staff person who asks about clients’ desire to become pregnant, use of contraception, and whether or not she has any questions or concerns about her sexual or reproductive health.

The findings from this survey document that only a minority of resettlement offices are directly helping refugee women and couples plan for their futures by planning their pregnancies and families, or linking them to SRH services. For example, only 15% of responding agencies provide written SRH information to clients, and only 5% had posters or signs providing this information. Only 41% of offices provide SRH content in classes or workshops, and just slightly more than 25% provide information specifically about family planning and contraceptive methods. While the majority of agencies refer clients for primary care and intend that refugees seek reproductive healthcare through that resource, only a small percentage of agencies refer clients to clinical resources specifically for reproductive healthcare. These findings highlight an oversight in the provision of care for refugee women, often times leaving the burden of seeking to understand, request, and access SRH services solely to women themselves. These findings point to a gap in services for refugee women, a gap that may have a detrimental effect on those women’s ability to set and reach their goals in a new community, such as educational, career, and well-being goals.

Refugee resettlement agencies cited a number of barriers to providing SRH information and referrals to clients, including the presence of multiple competing priorities, a lack of culturally appropriate sexual health materials to give to clients, and clients’ own attitudes and cultural beliefs about reproductive health. Some agencies felt that addressing reproductive health was outside the scope of resettlement services, while other agencies reported lack of time and resources as the key barrier to providing this service. However, having an unintended or mistimed pregnancy would be detrimental to a family’s ability to achieve economic self-sufficiency, so attending to clients’ SRH needs is a necessary part of supporting their resettlement.

The majority of respondents reported that their agency would or might be interested in improving their capacity to provide SRH information and referrals. The most commonly cited needs for offices to expand this capacity were written materials in multiple languages developed specifically for refugee clients and staff training on how to talk to clients about SRH. These findings point to the lack of on-site technical capacity at resettlement agencies to accurately and effectively formulate and share this information with women.

### Limitations

For this study, IRC affiliate offices were recruited through IRC’s national headquarters, whereas non-IRC offices were recruited without the participation of their sponsoring agency, which likely explains the differential response rate between non-IRC and IRC resettlement offices. The high response rate among IRC offices makes it likely that the survey results accurately reflect the practices and resources available at all IRC offices. The same cannot necessarily be said of the non-IRC offices; with the lower response rate from non-IRC offices, study authors cannot definitively assert that the responses are representative of all resettlement offices. Also, the survey instrument was not validated against other sources of information regarding the practices of refugee resettlement offices. Additionally, while the survey did assess which topics were covered in written materials and posters, too few respondents answered these questions to allow for any meaningful discussion of material content. Finally, this study examines the provision of SRH information and education from the perspective of service providers, and not the experiences of refugee women themselves.

## Recommendations

Recognizing that resettlement agencies may lack the capacity to meet the SRH needs of their clients due to limited resources, the following recommendations may be helpful for integrating SRH services into existing resettlement service delivery.

### Inform and educate clients

RRA staff inform and educate clients about a wide range of issues needed for their resettlement journey to live healthy and empowered lives. Staff can make a significant impact on refugee youth and women’s ability to make informed decisions and advocate for what they need and want in healthcare systems by offering precise information; presenting information in languages and formats that are accessible and appealing; and delivering information in ways that are nonjudgmental and compassionate. The easiest way to do this is to provide written materials in the form of handouts and brochures given to clients and posters prominently displayed in the office, including restrooms. This information should be available in the languages most commonly spoken by an agency’s clients. Workshops focused on birth control, planning a family, and seeking reproductive healthcare are also an important mechanism for educating clients. If RRAs do not have the capacity to have free-standing workshops, they can incorporate information and education into cultural orientation sessions or English language classes. The information refugee youth and women receive can support them in their practice of preventive SRH behaviors.

### Assess pregnancy intentions and refer to services

One of the best ways to engage clients in seeking SRH information is to assess their pregnancy intention. This refers to people’s stated ideas about whether and when to be pregnant with first or subsequent pregnancies. Asking refugee women and youth about whether they would like to be pregnant within the next 6 months can help RRA staff connect clients to the information, resources, and services they need and desire, including prenatal, abortion, and contraceptive care. Yet only 12% of offices said that they asked this simple question. These questions may be integrated into standard intake process, or incorporated as part of other screenings, e.g., mental health and gender-based violence.

For clients who do not want to become pregnant in the next year, referral to contraceptive services is a vital next step; however, while most agencies were aware of a service provider in their area, few made the direct referral. Staff should become familiar with local service provides in order to facilitate communication about client needs, including interpretation. This can put the client at greater ease before their appointment.

### Partnering with SRH care providers

Education, screening, and referral are good first steps but are often not sufficient to meet the range of pregnancy and contraceptive care needs that refugee women and youth have. Collaboration and partnerships between SRH providers and refugee resettlement agencies can fill important gaps and benefit both agency staff and clients. Partnerships can take many forms and agencies should choose the relationship that best helps clients overcome common challenges, such as being able to afford services; scheduling appointments through a live person and not an automated line; securing transportation to appointments; having interpretation during the appointment; and arranging for follow-up with the clinic. Good partnerships can offer reciprocal information sharing so that each partner can have a deep understanding of the services the other provides and how to access these services and reciprocal learning, so lessons learned are shared and documented and become the basis for the future direction of the partnership. Ideally, partnerships will also allow agencies to leverage funding in ways that can improve and expand contraceptive care and related services.

### Building the capacity of contraceptive care providers

Finally, the authors believe that RRAs are in a unique position to build the capacity of contraceptive care providers in their areas to better serve the needs of refugee women and youth. This important work will most likely take place in healthcare facilities with which RRAs have already established some degree of relationship, be it through referrals or partnership. RRA staff can offer insights about the refugee populations in their area that can help healthcare providers offer client-centered, trauma-informed, culturally and linguistically responsive care to refugee women and youth.

## Conclusions

Findings from this survey may assist with laying the groundwork for strengthening the capacity of US RRAs to integrate SRH services into programming and to support refugees’ ability to plan for their futures and to have intended pregnancies and families. The current landscape of capacity, established in part through this survey, will inform the cross-organizational effort by IRC and AFY to pilot programs aimed at increasing access to SRH education and services for refugee clients. Further, additional research into the experiences of refugee women themselves would contribute greatly to an understanding of their needs for service provision.

## Supporting information

S1 Survey Contains the survey instrument. (PDF)

S1 Data Contains the survey data. (PDF)

S1 Checklist Contains the checklist of guidelines from Standards for Reporting Qualitative Research, as published in the journal Academic Medicine in September of 2014 [7]. (PDF)

## Abbreviations

AFY: Advocates for Youth
IRC: International Rescue Committee
RRA: refugee resettlement agency
SRH: sexual and reproductive health
