# Supplementary material for: Sexual and reproductive health information and referrals for resettled refugee women: A survey of resettlement agencies in the United States
Source: PLoS Med. 2021 May 3;18(5):e1003579. doi: 10.1371/journal.pmed.1003579 (PMC8092785; doi:10.1371/journal.pmed.1003579)
Supplement: S1 Survey — (PDF) [file pmed.1003579.s001.pdf]

Resettlement Agency Reproductive Health Survey

Thank you for participating in this survey. This survey is a project of Advocates for Youth, with assistance from Harvard T.H. Chan School of Public Health. The goal of the survey is to understand what resources are available at refugee resettlement agencies in the United States to assist refugees in accessing reproductive and sexual health information, education, and clinical care. The survey is part of a broader project to strengthen the capacity of refugee resettlement agencies to provide this information and to support refugees' ability to plan for their futures and to avoid unintended pregnancies. Survey results will be used to inform the development of resources for refugees and for resettlement agencies. Results may be published and/or presented at relevant conferences; however, no identifying information about specific respondents or agencies will be shared.

In order to understand refugee resettlement agencies' current capacity to provide reproductive health information, education, and resources, we request that one person from your office complete the survey, preferably someone who is familiar with the range of resources available to refugees. In appreciation for your time and attention, each office that completes the survey will be entered into a drawing for a prize of $500 that will be given to the agency office, to be used in any way the agency desires.

In addition, please note that the survey does ask for your name and contact information. This is being collected so that we can follow up with you to offer resources and support in the future. If you would prefer not to give your name and contact information, you may leave those questions blank. Note that you can still enter the drawing without leaving your contact information - the drawing entry is a separate form and is not linked to your survey responses in any way.

If you have questions or comments about this survey, please contact Tonya Katcher at tonya@advocatesforyouth.org.

Resettlement Agency Reproductive Health Survey

1. What organization or organizations is your resettlement office affiliated with? (Check all that apply)

- [ ] Church World Service
- [ ] Ethiopian Community Development Council
- [ ] Episcopal Migration Ministries
- [ ] Hebrew Immigrant Aid Society
- [ ] International Rescue Committee
- [ ] Lutheran Immigration and Refugee Service
- [ ] U.S. Committee for Refugees and Immigrants
- [ ] United States Conference of Catholic Bishops/Migration and Refugee Services
- [ ] World Relief

2. What is your job title/role at your agency?

3. Does your agency offer written materials (such as pamphlets or handouts) on sexual and reproductive health for clients?

- ◯ Yes
- ◯ No

4. What topics are covered in your agency's written materials? (Check all that apply)

- [ ] The reproductive system and how pregnancy happens
- [ ] Family planning or child spacing
- [ ] Birth control methods (including the pill, shot, IUD, etc)
- [ ] STI prevention, testing, and treatment
- [ ] Condom use
- [ ] Healthy relationships/communication/consent
- [ ] Resources for pregnant women
- [ ] How and where to access clinical reproductive health services
- [ ] Preventive care for sexual and reproductive health (cancer screenings, immunizations, etc)
- [ ] Sexual and reproductive health for adolescents and young adults
- [ ] Other (please specify)

5. Are these materials available in multiple languages?

- ◯ No, they are only available in English
- ◯ They are available in English plus 1-2 other languages
- ◯ They are available in English plus 3 or more other languages

6. Are any of these materials developed to be accessible to clients with low literacy?

- ◯ Yes
- ◯ No

7. Does your agency have any posters or displayed signs that provide sexual and reproductive health information?

- Yes
- No

8. What topics are included on posters or displayed signs in your agency? (Check all that apply)

- [ ] The reproductive system and how pregnancy happens
- [ ] Family planning or child spacing
- [ ] Birth control methods (including the pill, shot, IUD, etc)
- [ ] STI prevention, testing, and treatment
- [ ] Condom use
- [ ] Healthy relationships/communication/consent
- [ ] Resources for pregnant women
- [ ] How and where to access clinical reproductive health services
- [ ] Preventive care for sexual and reproductive health (cancer screenings, immunizations, etc)
- [ ] Sexual and reproductive health for adolescents and young adults
- [ ] Other (please specify)

9. Are these materials available in multiple languages?

- No, they are only available in English
- They are available in English plus 1-2 other languages
- They are available in English plus 3 or more other languages

10. Are any of these materials developed to be accessible to clients with low literacy?

- Yes
- No

11. Does your agency office offer any classes or workshops that cover reproductive health topics?

- ◯ Yes
- ◯ No

12. What does your agency offer? (Check all that apply)

- [ ] A workshop (in a single session)
- [ ] A multi-session class devoted to reproductive health
- [ ] Reproductive health content integrated into a broader course (such as cultural orientation)
- [ ] Other (please specify):

13. What topics are covered in the educational offerings? (Check all that apply)

- [ ] The reproductive system and how pregnancy happens
- [ ] Family planning or child spacing
- [ ] Birth control methods (including the pill, shot, IUD, etc)
- [ ] STI prevention, testing, and treatment
- [ ] Condom use
- [ ] Healthy relationships/communication/consent
- [ ] Resources for pregnant women
- [ ] How and where to access clinical reproductive health services
- [ ] Preventive care for sexual and reproductive health (cancer screenings, immunizations, etc)
- [ ] Sexual and reproductive health for adolescents and young adults
- [ ] Other (please specify)

14. Are classes or workshops on reproductive health mandatory or voluntary?

- Mandatory
- Voluntary

15. Are classes or workshops on reproductive health separated by gender?

- Always
- Usually
- Sometimes
- Rarely
- Never

16. How frequently are classes or workshops on reproductive health offered?

- Every month
- 4-6 times per year
- 1-3 times per year
- Infrequently/as needed

17. Does your agency office partner with any other organization to provide classes or workshops that cover reproductive health topics?

- Yes
- No

18. If yes, please list:

19. Does your agency have a health educator on staff?

- ◯ Yes
- ◯ No

20. Does that person provide information about any of the following topics? (Check all that apply)

- ☐ The reproductive system and how pregnancy happens
- ☐ Family planning or child spacing
- ☐ Birth control methods (including the pill, shot, IUD, etc)
- ☐ STI prevention, testing, and treatment
- ☐ Condom use
- ☐ Healthy relationships/communication/consent
- ☐ Resources for pregnant women
- ☐ How and where to access clinical reproductive health services
- ☐ Preventive care for sexual and reproductive health (cancer screenings, immunizations, etc)
- ☐ Sexual and reproductive health for adolescents and young adults
- ☐ Other (please specify)

21. Does the health educator meet with women individually, without other family members present?

- ○ Always
- ○ Usually
- ○ Sometimes
- ○ Rarely
- ○ Never

22. Do you routinely ask women clients, upon intake to your agency, if they want to become pregnant in the next year?

- Always
- Usually
- Sometimes
- Rarely
- Never

23. Are there clinics that are accessible from your agency’s location where women can receive sexual and reproductive health services?

- ○ Yes
- ○ No
- ○ I don't know

24. Do you refer clients to a clinic for reproductive health services, including contraception and pre-conception care?

- ○ Yes, we refer clients specifically for reproductive healthcare
- ○ Yes, we refer clients for primary care, and intend that they receive reproductive healthcare from their primary provider, if needed
- ○ No, we do not refer clients to clinics

25. Do you provide any additional assistance to clients who wish to obtain reproductive health services? (Check all that apply)

- [ ] Assistance calling and making an appointment
- [ ] Transportation to the clinic for appointments
- [ ] Interpretation services for clinical appointments
- [ ] Other (please specify)
- [ ] Assistance obtaining a medication or filling a prescription
- [ ] Assistance obtaining health insurance or paying for healthcare services

26. In your opinion, what are the barriers your office faces in ensuring women clients receive sexual and reproductive health information and services? (Check all that apply)

- [ ] Lack of time/too many competing priorities
- [ ] Staff members' lack of knowledge or skills discussing reproductive health
- [ ] Lack of culturally and linguistically appropriate materials about reproductive health to provide to clients
- [ ] Clients' cultural backgrounds, attitudes, and beliefs about reproductive health
- [ ] Clients' lack of knowledge about reproductive health
- [ ] Staff members' cultural backgrounds, attitudes, and beliefs about reproductive health
- [ ] Office policies and culture discourage discussing reproductive health topics
- [ ] Other (please specify)

27. In your opinion, do you think that your agency office would be interested in increasing its capacity to provide sexual and reproductive health information and referrals to clients?

- Yes
- Maybe
- No
- I don't know

28. What resources do you think would be most helpful to your agency office to increase your capacity to provide sexual and reproductive health information and referrals to clients? (Check all that apply)

- Staff training about sexual and reproductive healthcare
- Staff training about birth control
- Staff training about how to talk to clients about sexual and reproductive health
- Written materials (handouts, pamphlets) for clients with sexual health information
- Other (please specify)

Resettlement Agency Reproductive Health Survey

Thank you for taking the time to complete this survey. We greatly value your knowledge and experience and the work that you do! We would like the opportunity to follow up with you and to offer resources and training. However, even if you prefer not to provide your contact information, your agency can be entered into the drawing for a $500 donation by clicking on the link on the following page.

29. Contact information:

**Name**	
**Company**	
**Address**	
**Address 2**	
**City/Town**	
**State/Province**	-- select state --
**ZIP/Postal Code**	
**Email Address**	
**Phone Number**	

Resettlement Agency Reproductive Health Survey

Thank you again for taking this survey. To enter your agency in the drawing for a donation of $500, please follow the link below.

Resettlement Agency Drawing

If you have questions or comments about this survey, please contact Tonya Katcher, at Advocates for Youth, at tonya@advocatesforyouth.org
