# Supplementary material for: Sexual and reproductive health information and referrals for resettled refugee women: A survey of resettlement agencies in the United States
Source: PLoS Med. 2021 May 3;18(5):e1003579. doi: 10.1371/journal.pmed.1003579 (PMC8092785; doi:10.1371/journal.pmed.1003579)
Supplement: S1 Data — (PDF) [file pmed.1003579.s002.pdf]

Q1 What organization or organizations is your resettlement office affiliated with? (Check all that apply)

Answered: 100 Skipped: 0

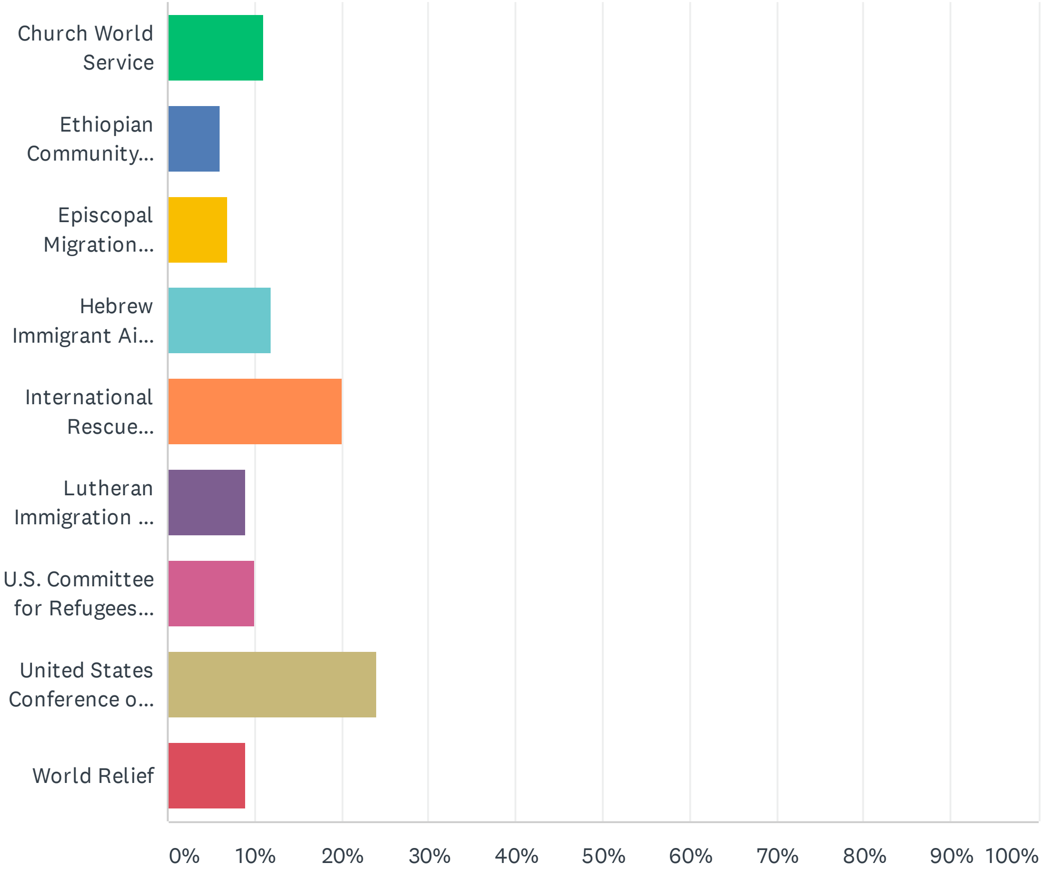

ANSWER CHOICES	RESPONSES	
Church World Service	11.00%	11
Ethiopian Community Development Council	6.00%	6
Episcopal Migration Ministries	7.00%	7
Hebrew Immigrant Aid Society	12.00%	12
International Rescue Committee	20.00%	20
Lutheran Immigration and Refugee Service	9.00%	9
U.S. Committee for Refugees and Immigrants	10.00%	10
United States Conference of Catholic Bishops/Migration and Refugee Services	24.00%	24
World Relief	9.00%	9
Total Respondents: 100		

Q2 What is your job title/role at your agency?

Answered: 95 Skipped: 5

#	RESPONSES	DATE
1	Wellness and Gender Equity Supervisor	9/13/2018 2:55 PM
2	Gender Equity and Family Wellness Coordinator	9/13/2018 2:50 PM
3	Medical Case Manager	9/13/2018 2:46 PM
4	Senior Health Liaison	9/13/2018 2:40 PM
5	Resettlement Coordinator	9/13/2018 2:35 PM
6	Executive Director	9/13/2018 1:51 PM
7	Deputy Director	9/13/2018 1:47 PM
8	Executive Director	9/13/2018 1:42 PM
9	Resettlement Coordinator	9/13/2018 1:35 PM
10	Resettlement and Anti-Trafficking Manager; Acting Site Manager	9/13/2018 1:31 PM
11	Resettlement Manager	9/11/2018 4:51 PM
12	Site Manager	9/11/2018 4:47 PM
13	Health and Gender Senior Program Manager	9/11/2018 4:22 PM
14	casework supervisor	9/11/2018 4:18 PM
15	Health Program Coordinator	9/11/2018 4:14 PM
16	Health and Wellness Team	9/11/2018 2:58 PM
17	Deputy Director	9/11/2018 2:52 PM
18	Resettlement Director	9/11/2018 2:35 PM
19	Resettlement Director - Abilene	9/11/2018 2:18 PM
20	Director of Resettlement Services	6/19/2018 8:54 AM
21	Program Manager	6/14/2018 6:13 PM
22	Health Access Manager	6/14/2018 4:30 PM
23	Interim Program Manager	6/13/2018 2:50 PM
24	Resettlement Coordinator	6/13/2018 1:25 PM
25	Refugee medical coordinator	6/13/2018 2:20 AM
26	Reception and Placement Coordinator	6/12/2018 7:17 PM
27	Director	6/12/2018 4:20 PM
28	Resettlement Director	6/12/2018 3:36 PM
29	Program Manager	6/12/2018 3:22 PM
30	Director	6/12/2018 3:03 PM
31	Resettlement Director	6/12/2018 2:58 PM
32	Refugee Health Care Coordinator	6/12/2018 9:21 AM
33	Director of Family Services	6/11/2018 4:52 PM
34	Senior Ongoing Services Coordinator, intensive case management and social adjustment services	6/8/2018 3:04 PM
35	CEO	6/8/2018 11:49 AM
36	Senior Program Coordinator	6/8/2018 11:48 AM
37	Executive Director	6/8/2018 11:12 AM

38	Executive Director	6/8/2018 9:32 AM
39	Director, Refugee and Immigrant Services	6/8/2018 9:21 AM
40	Director	6/7/2018 6:04 PM
41	Program Manager	6/7/2018 5:52 PM
42	Healthcare Specialist/medical case manager	6/7/2018 1:59 PM
43	community outreach coordinator	6/7/2018 1:44 PM
44	Program Director	6/7/2018 1:13 PM
45	Director, Refugee Arrival Services	6/7/2018 12:53 PM
46	Youth Program Manager	6/7/2018 12:19 PM
47	Director/SRC	6/7/2018 11:46 AM
48	Director of Social Services	6/7/2018 11:39 AM
49	R&P COORDINATOR	6/7/2018 11:38 AM
50	CEO	6/7/2018 11:32 AM
51	director of new american integration	6/7/2018 11:26 AM
52	VP of Immigration and Migration Services	6/7/2018 11:24 AM
53	Director	6/7/2018 11:21 AM
54	Director of REsettlement	6/7/2018 10:17 AM
55	Refugee Resettlement program Director	6/4/2018 10:13 AM
56	Director	6/4/2018 9:39 AM
57	Intensive Case Manager	6/4/2018 8:17 AM
58	Resettlement Director	6/1/2018 5:34 PM
59	Senior Program Director	6/1/2018 5:33 PM
60	Recettlement Program Coordinator	6/1/2018 4:32 PM
61	Director	6/1/2018 2:58 PM
62	Education & Employment Program Director	6/1/2018 2:31 PM
63	Department Direcor of Refugee and Immigration Services	6/1/2018 2:04 PM
64	Program Director	6/1/2018 1:38 PM
65	Resettlement Director	6/1/2018 12:36 PM
66	R&P Program Manager	6/1/2018 12:18 PM
67	Office Director	6/1/2018 12:15 PM
68	Director	6/1/2018 11:51 AM
69	Director	6/1/2018 11:37 AM
70	Area Director	5/10/2018 4:13 PM
71	Resettlement Coordinator	5/9/2018 5:33 PM
72	Coordinator/Director	5/4/2018 3:28 PM
73	Supervisor/immigration and Refugee Department	5/4/2018 1:53 PM
74	Program Manager	5/4/2018 1:37 PM
75	Executive Director	5/2/2018 5:27 PM

76	CEO	5/2/2018 4:48 PM
77	Program Coordinator for Resettlement	5/2/2018 11:09 AM
78	Program Manager	5/1/2018 3:59 PM
79	Resettlement director	5/1/2018 3:42 PM
80	Senior Case Manager	5/1/2018 2:59 PM
81	Refugee Resettlement Director	4/30/2018 12:51 PM
82	Director of Refugee Services	4/27/2018 1:44 PM
83	Resettlement Specialist	4/27/2018 9:54 AM
84	Senior Resettlement Specialist	4/24/2018 4:09 PM
85	Administrative Assistant	4/24/2018 1:15 PM
86	Program Director	4/24/2018 9:43 AM
87	Resettlement Assistant	4/24/2018 8:54 AM
88	Health and Social Services Program Manager	4/23/2018 4:23 PM
89	Director	4/23/2018 2:31 PM
90	Program Manager	4/23/2018 1:56 PM
91	Resettlement Director	4/23/2018 1:40 PM
92	Director of Case Management	4/23/2018 12:58 PM
93	Team Manager: Resettlement Services	4/23/2018 12:55 PM
94	Case Management Coordinator	4/23/2018 12:26 PM
95	Resettlement Manager	4/18/2018 4:33 PM

Q3 Does your agency offer written materials (such as pamphlets or handouts) on sexual and reproductive health for clients?

Answered: 94   Skipped: 6

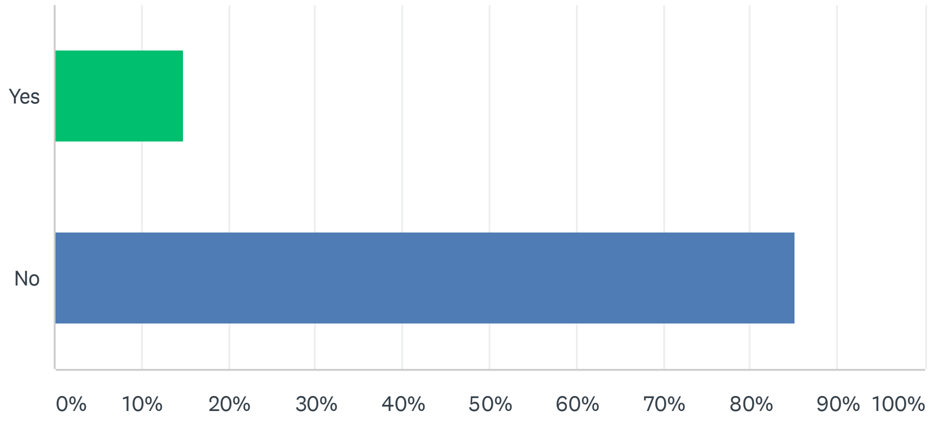

ANSWER CHOICES	RESPONSES	
Yes	14.89%	14
No	85.11%	80
TOTAL		94

Q4 What topics are covered in your agency's written materials? (Check all that apply)

Answered: 12 Skipped: 88

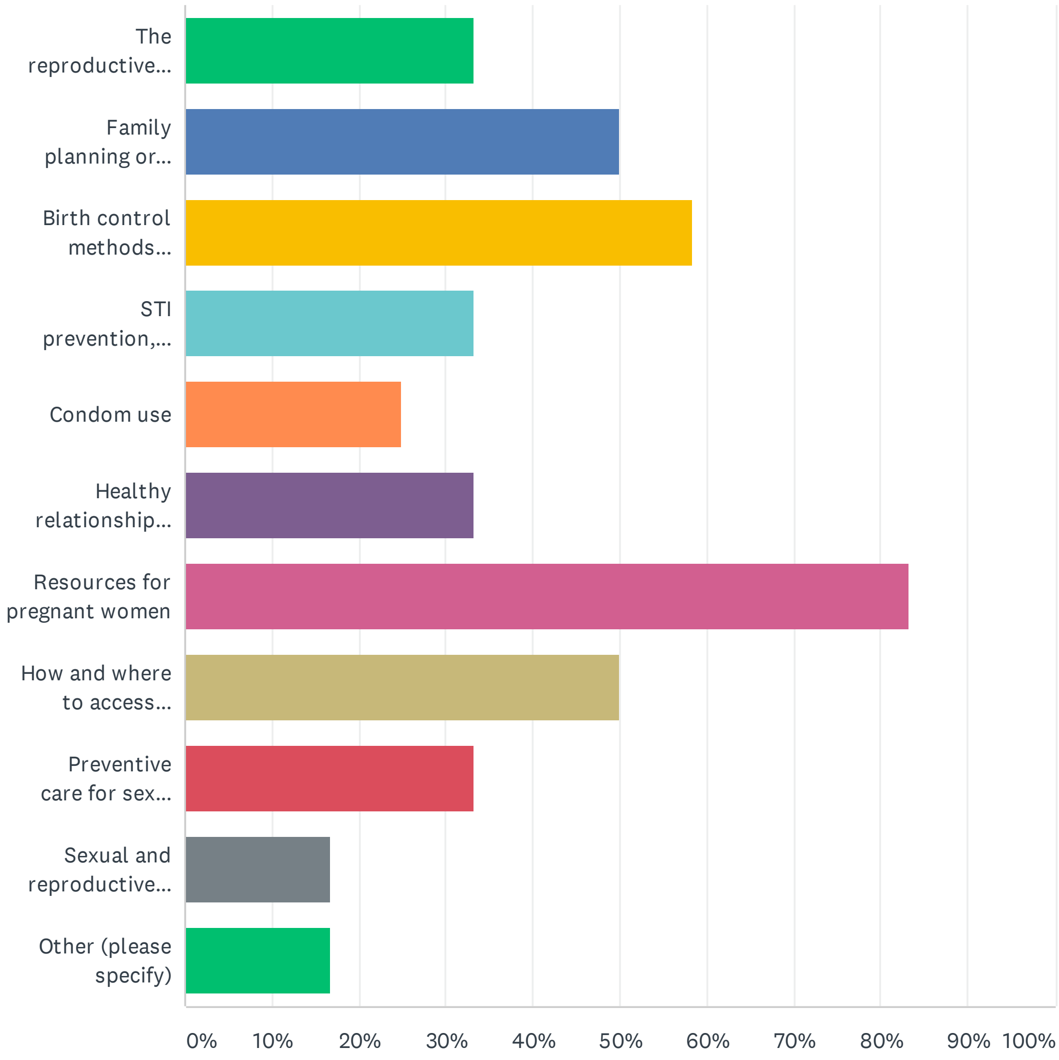

ANSWER CHOICES	RESPONSES	
The reproductive system and how pregnancy happens	33.33%	4
Family planning or child spacing	50.00%	6
Birth control methods (including the pill, shot, IUD, etc)	58.33%	7
STI prevention, testing, and treatment	33.33%	4
Condom use	25.00%	3
Healthy relationships/communication/consent	33.33%	4
Resources for pregnant women	83.33%	10
How and where to access clinical reproductive health services	50.00%	6
Preventive care for sexual and reproductive health (cancer screenings, immunizations, etc)	33.33%	4
Sexual and reproductive health for adolescents and young adults	16.67%	2
Other (please specify)	16.67%	2
Total Respondents: 12		

Q5 Are these materials available in multiple languages?

Answered: 12 Skipped: 88

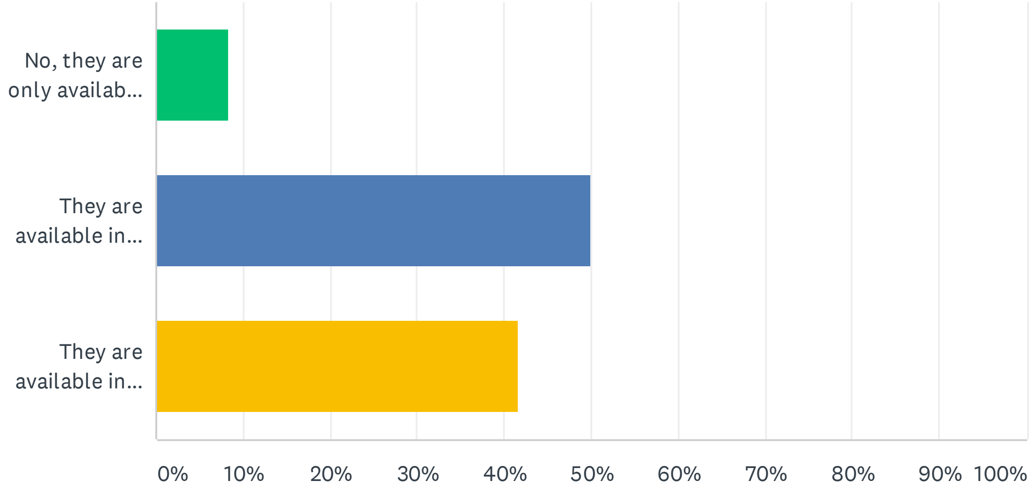

ANSWER CHOICES	RESPONSES	
No, they are only available in English	8.33%	1
They are available in English plus 1-2 other languages	50.00%	6
They are available in English plus 3 or more other languages	41.67%	5
TOTAL		12

Q6 Are any of these materials developed to be accessible to clients with low literacy?

Answered: 12 Skipped: 88

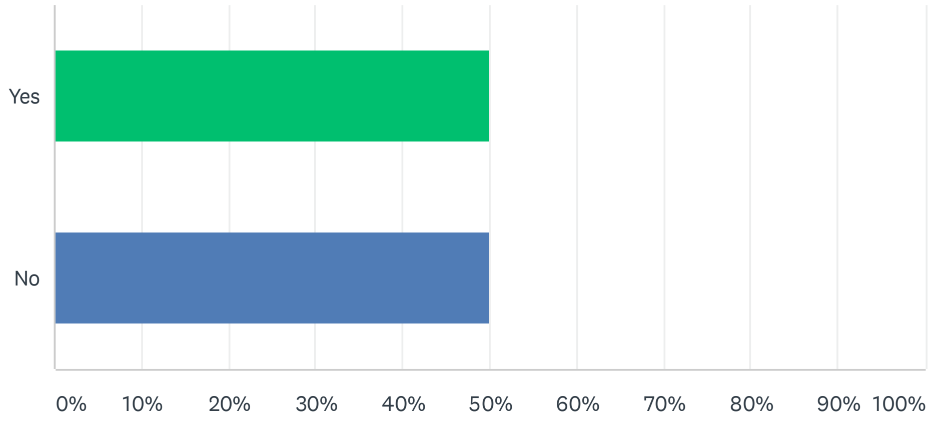

ANSWER CHOICES	RESPONSES	
Yes	50.00%	6
No	50.00%	6
TOTAL		12

Q7 Does your agency have any posters or displayed signs that provide sexual and reproductive health information?

Answered: 91 Skipped: 9

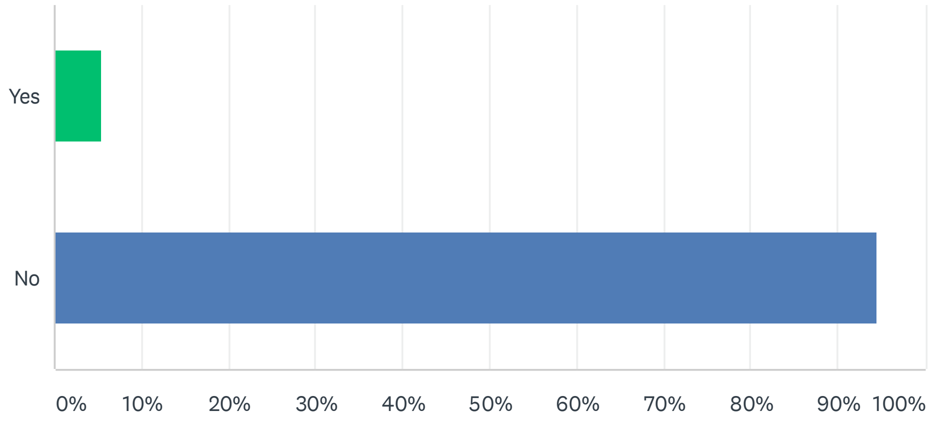

ANSWER CHOICES	RESPONSES	
Yes	5.49%	5
No	94.51%	86
TOTAL		91

Q8 What topics are included on posters or displayed signs in your agency? (Check all that apply)

Answered: 5 Skipped: 95

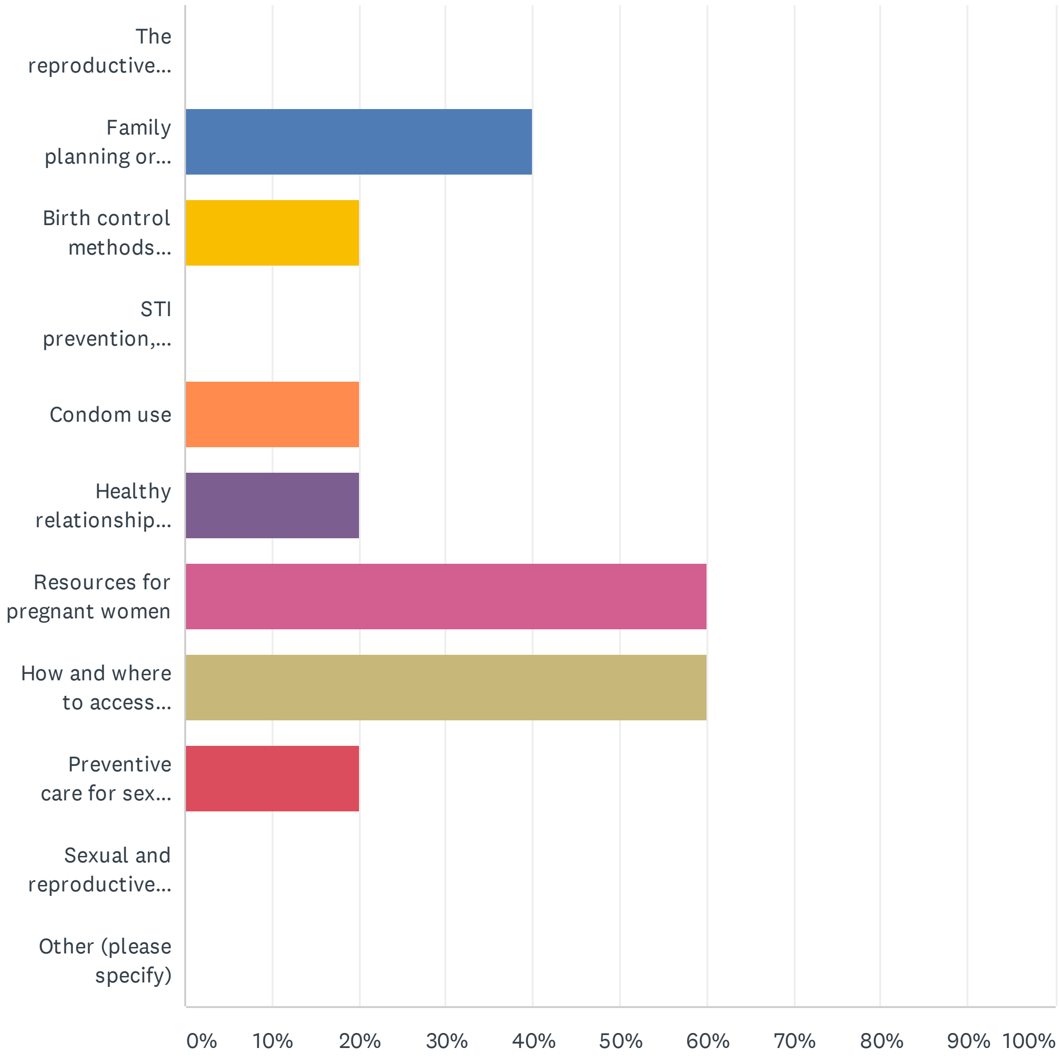

ANSWER CHOICES	RESPONSES	
The reproductive system and how pregnancy happens	0.00%	0
Family planning or child spacing	40.00%	2
Birth control methods (including the pill, shot, IUD, etc)	20.00%	1
STI prevention, testing, and treatment	0.00%	0
Condom use	20.00%	1
Healthy relationships/communication/consent	20.00%	1
Resources for pregnant women	60.00%	3
How and where to access clinical reproductive health services	60.00%	3
Preventive care for sexual and reproductive health (cancer screenings, immunizations, etc)	20.00%	1
Sexual and reproductive health for adolescents and young adults	0.00%	0
Other (please specify)	0.00%	0
Total Respondents: 5		

Q9 Are these materials available in multiple languages?

Answered: 5   Skipped: 95

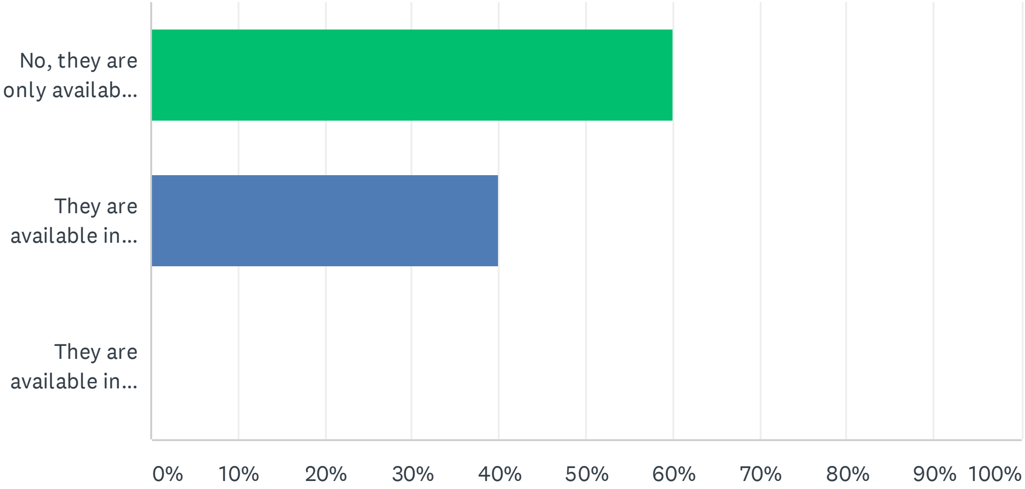

ANSWER CHOICES	RESPONSES	
No, they are only available in English	60.00%	3
They are available in English plus 1-2 other languages	40.00%	2
They are available in English plus 3 or more other languages	0.00%	0
TOTAL		5

Q10 Are any of these materials developed to be accessible to clients with low literacy?

Answered: 5 Skipped: 95

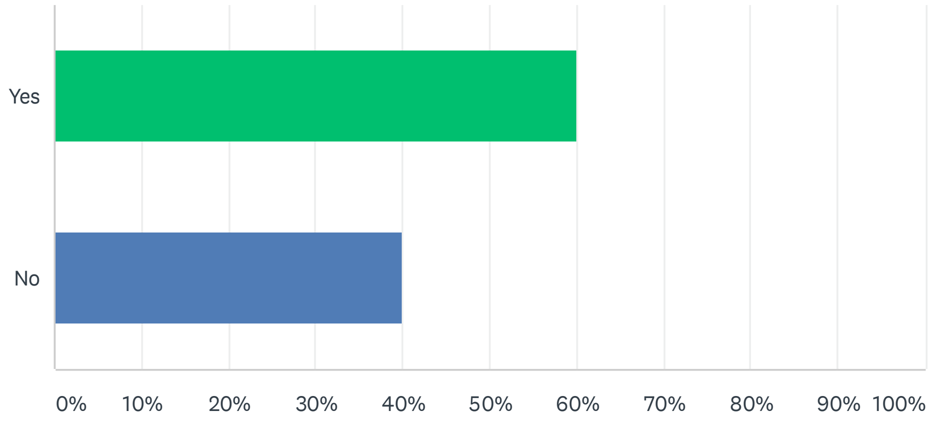

ANSWER CHOICES	RESPONSES	
Yes	60.00%	3
No	40.00%	2
TOTAL		5

Q11 Does your agency office offer any classes or workshops that cover reproductive health topics?

Answered: 91 Skipped: 9

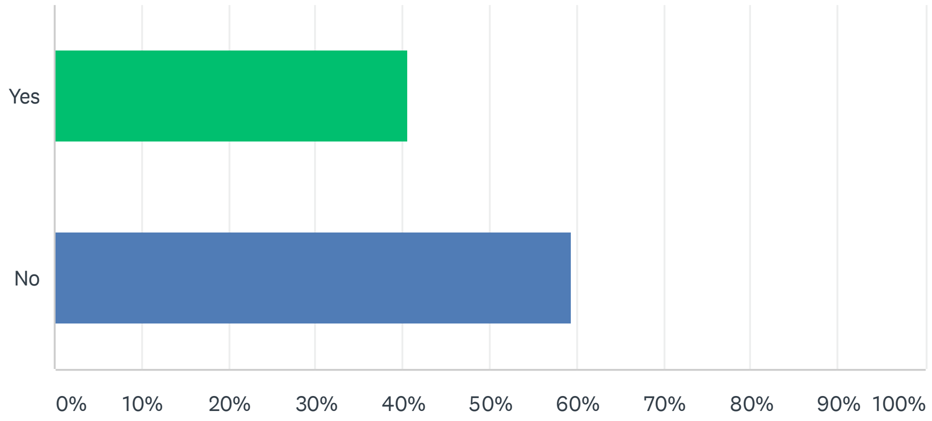

ANSWER CHOICES	RESPONSES	
Yes	40.66%	37
No	59.34%	54
TOTAL		91

Q12 What does your agency offer? (Check all that apply)

Answered: 35 Skipped: 65

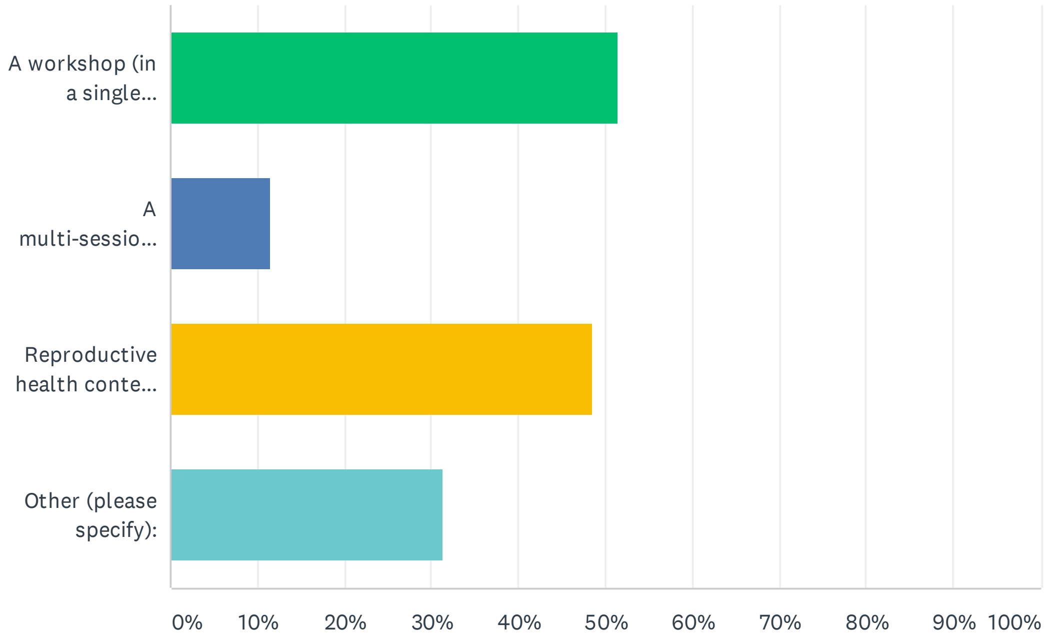

ANSWER CHOICES	RESPONSES	
A workshop (in a single session)	51.43%	18
A multi-session class devoted to reproductive health	11.43%	4
Reproductive health content integrated into a broader course (such as cultural orientation)	48.57%	17
Other (please specify):	31.43%	11
Total Respondents: 35		

Q13 What topics are covered in the educational offerings? (Check all that apply)

Answered: 34 Skipped: 66

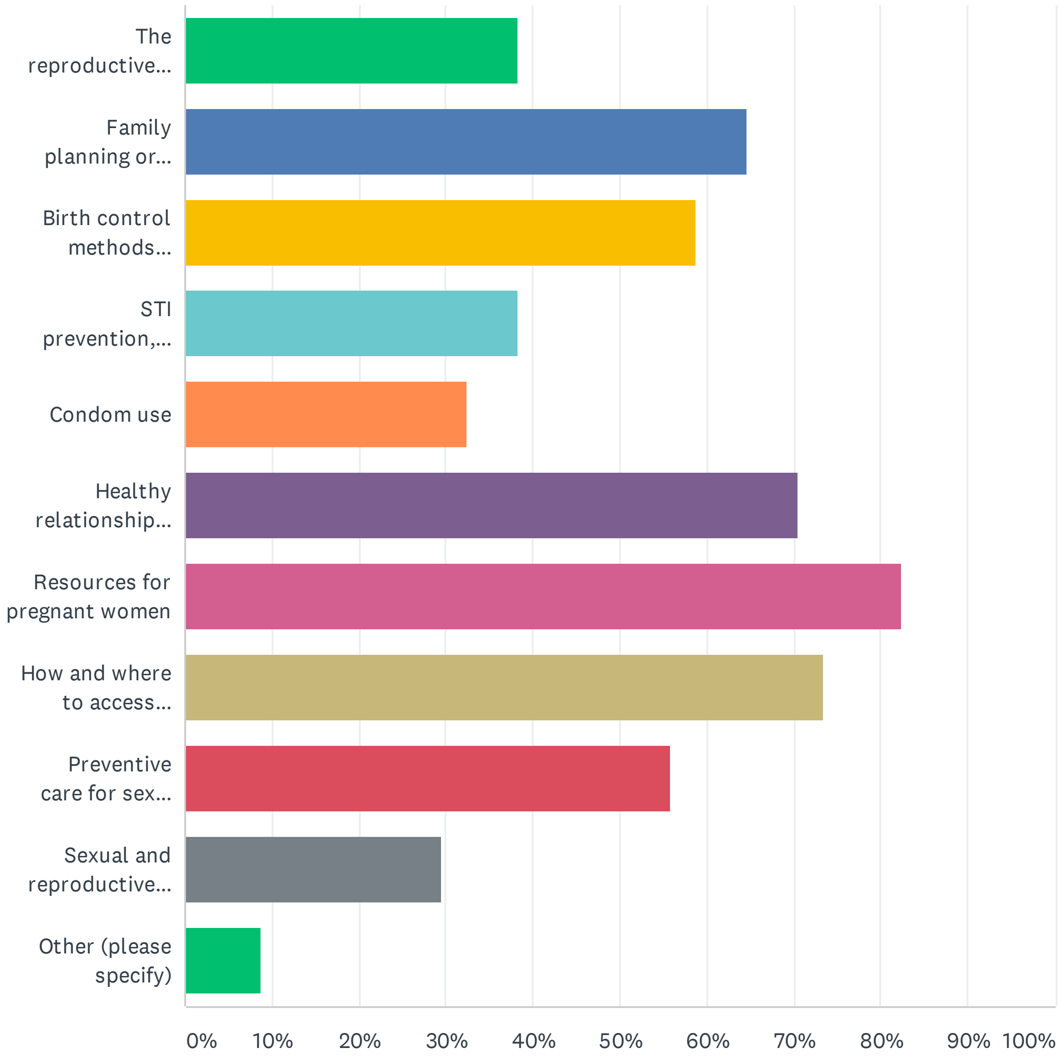

ANSWER CHOICES	RESPONSES	
The reproductive system and how pregnancy happens	38.24%	13
Family planning or child spacing	64.71%	22
Birth control methods (including the pill, shot, IUD, etc)	58.82%	20
STI prevention, testing, and treatment	38.24%	13
Condom use	32.35%	11
Healthy relationships/communication/consent	70.59%	24
Resources for pregnant women	82.35%	28
How and where to access clinical reproductive health services	73.53%	25
Preventive care for sexual and reproductive health (cancer screenings, immunizations, etc)	55.88%	19
Sexual and reproductive health for adolescents and young adults	29.41%	10
Other (please specify)	8.82%	3
Total Respondents: 34		

Q14 Are classes or workshops on reproductive health mandatory or voluntary?

Answered: 34 Skipped: 66

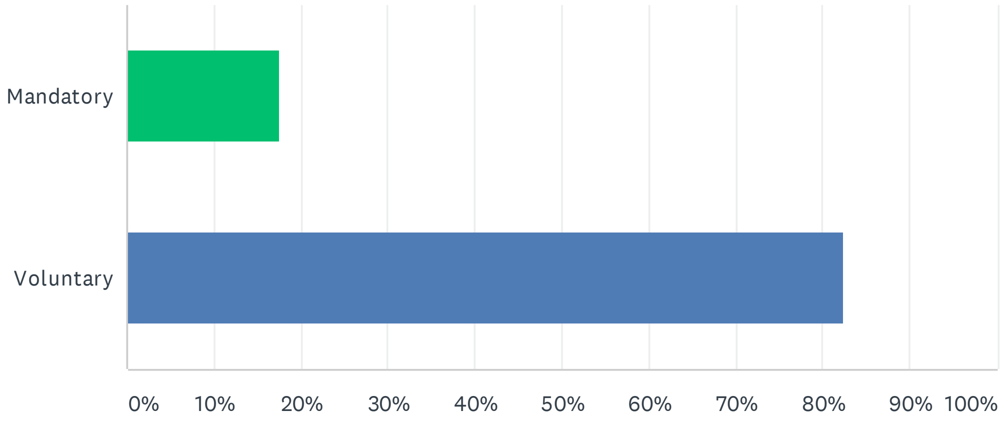

ANSWER CHOICES	RESPONSES	
Mandatory	17.65%	6
Voluntary	82.35%	28
TOTAL		34

Q15 Are classes or workshops on reproductive health separated by gender?

Answered: 35 Skipped: 65

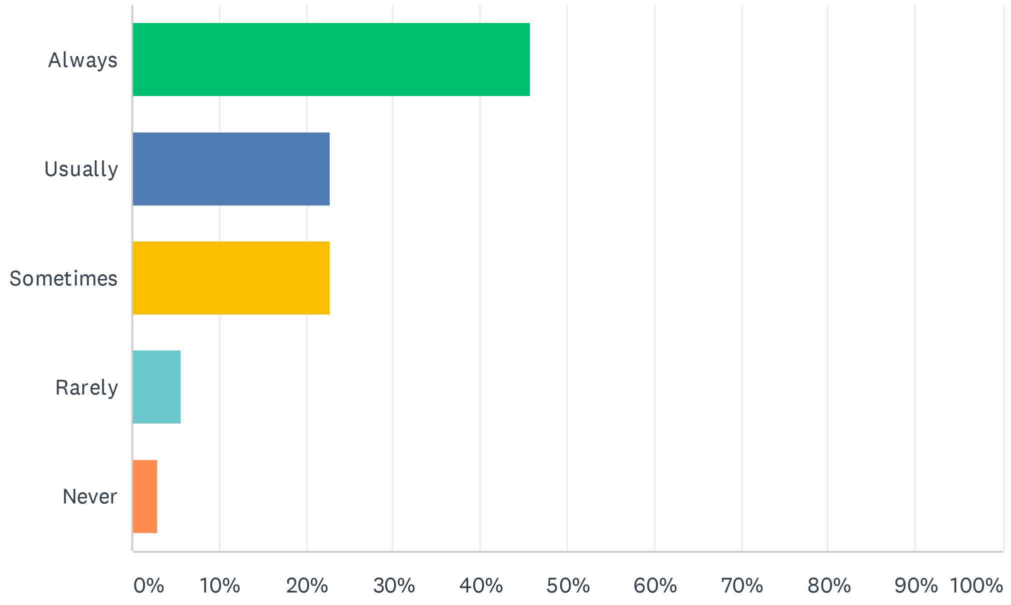

ANSWER CHOICES	RESPONSES	
Always	45.71%	16
Usually	22.86%	8
Sometimes	22.86%	8
Rarely	5.71%	2
Never	2.86%	1
TOTAL		35

Q16 How frequently are classes or workshops on reproductive health offered?

Answered: 35 Skipped: 65

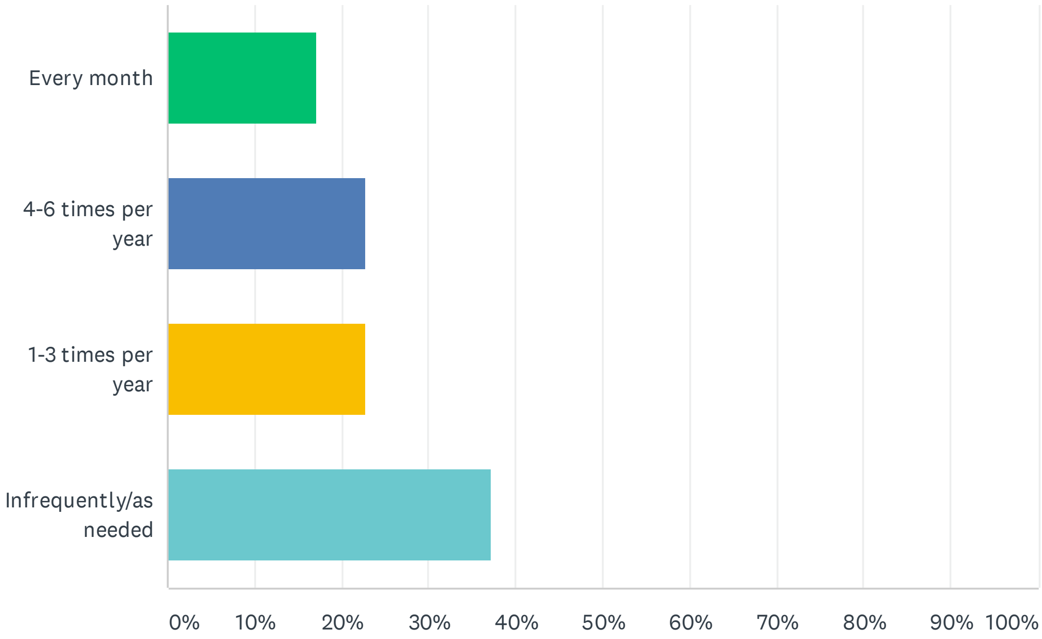

ANSWER CHOICES	RESPONSES	
Every month	17.14%	6
4-6 times per year	22.86%	8
1-3 times per year	22.86%	8
Infrequently/as needed	37.14%	13
TOTAL		35

Q17 Does your agency office partner with any other organization to provide classes or workshops that cover reproductive health topics?

Answered: 86 Skipped: 14

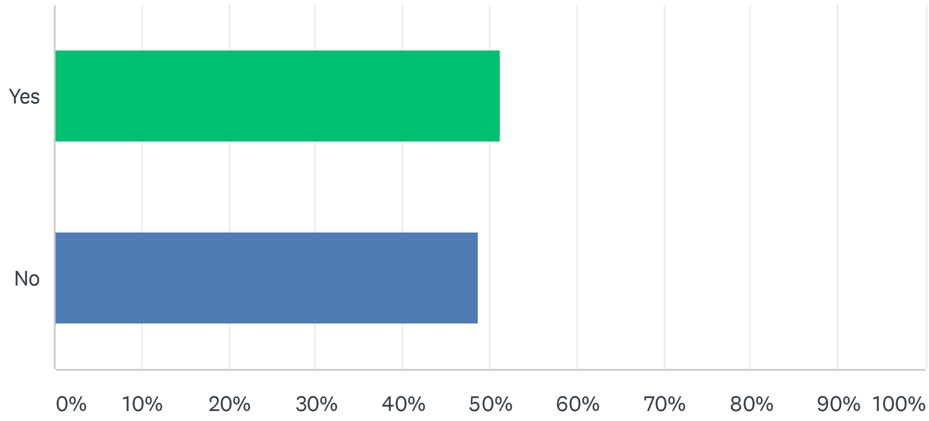

ANSWER CHOICES	RESPONSES	
Yes	51.16%	44
No	48.84%	42
TOTAL		86

Q18 If yes, please list:

Answered: 41 Skipped: 59

Q19 Does your agency have a health educator on staff?

Answered: 86 Skipped: 14

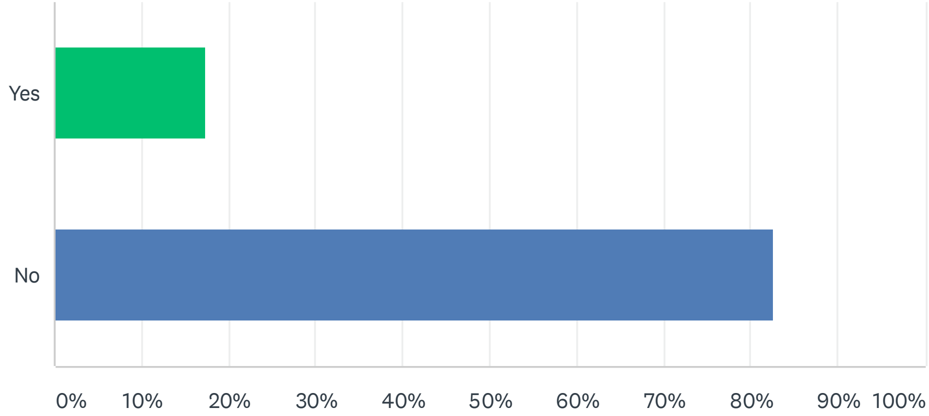

ANSWER CHOICES	RESPONSES	
Yes	17.44%	15
No	82.56%	71
TOTAL		86

Q20 Does that person provide information about any of the following topics? (Check all that apply)

Answered: 16 Skipped: 84

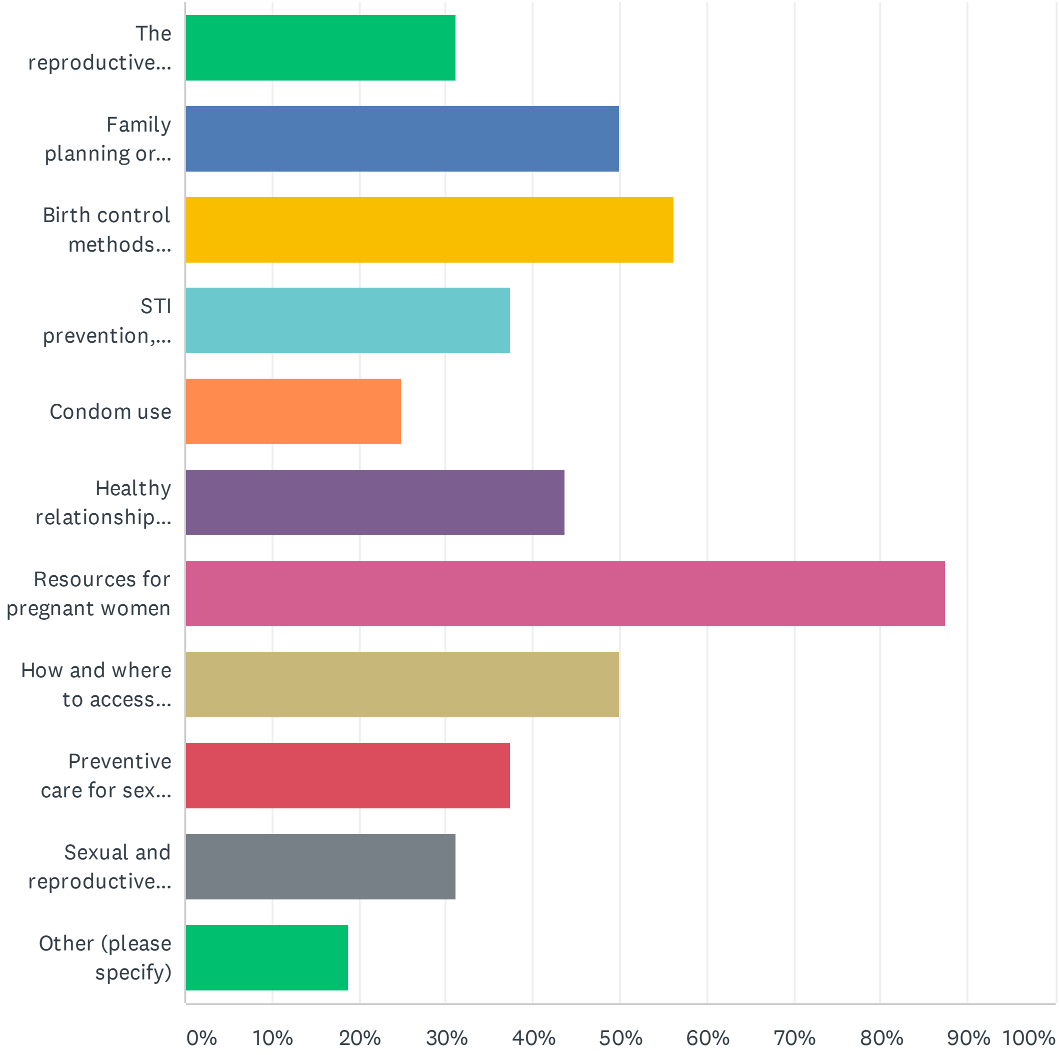

ANSWER CHOICES	RESPONSES	
The reproductive system and how pregnancy happens	31.25%	5
Family planning or child spacing	50.00%	8
Birth control methods (including the pill, shot, IUD, etc)	56.25%	9
STI prevention, testing, and treatment	37.50%	6
Condom use	25.00%	4
Healthy relationships/communication/consent	43.75%	7
Resources for pregnant women	87.50%	14
How and where to access clinical reproductive health services	50.00%	8
Preventive care for sexual and reproductive health (cancer screenings, immunizations, etc)	37.50%	6
Sexual and reproductive health for adolescents and young adults	31.25%	5
Other (please specify)	18.75%	3
Total Respondents: 16		

Q21 Does the health educator meet with women individually, without other family members present?

Answered: 15 Skipped: 85

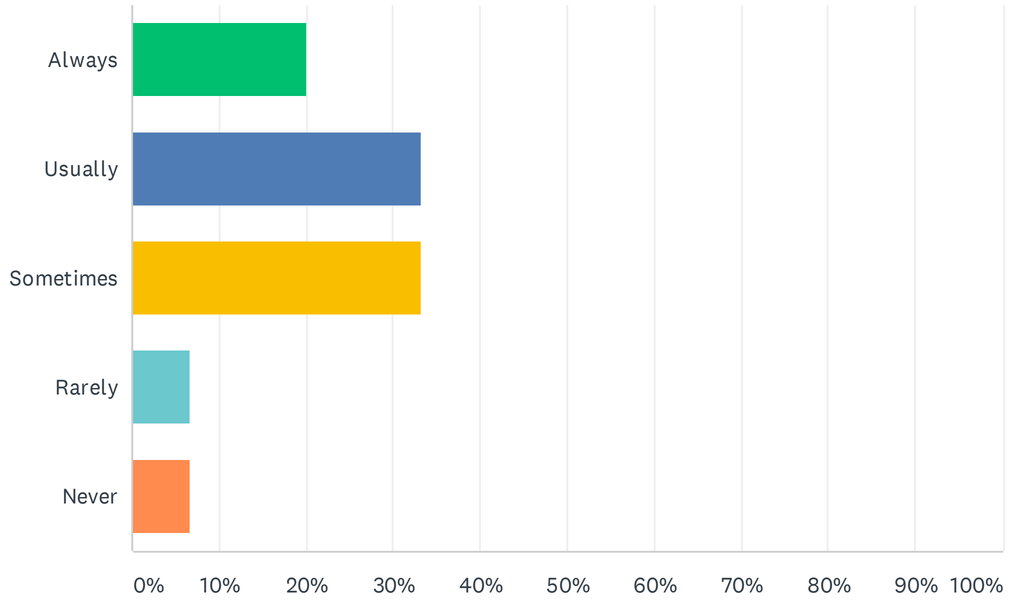

ANSWER CHOICES	RESPONSES	
Always	20.00%	3
Usually	33.33%	5
Sometimes	33.33%	5
Rarely	6.67%	1
Never	6.67%	1
TOTAL		15

Q22 Do you routinely ask women clients, upon intake to your agency, if they want to become pregnant in the next year?

Answered: 84   Skipped: 16

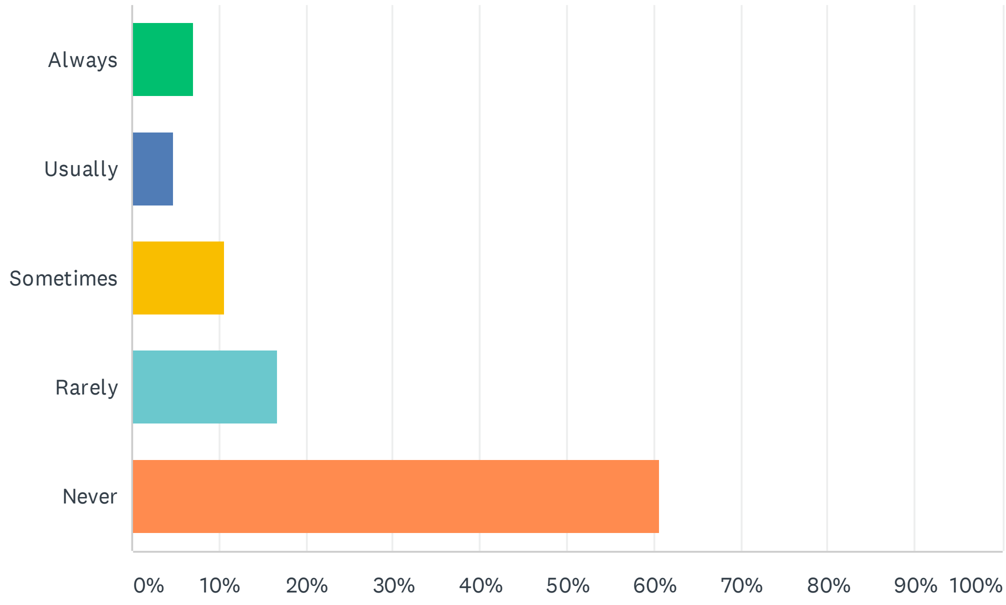

ANSWER CHOICES	RESPONSES	
Always	7.14%	6
Usually	4.76%	4
Sometimes	10.71%	9
Rarely	16.67%	14
Never	60.71%	51
TOTAL		84

Q23 Are there clinics that are accessible from your agency’s location where women can receive sexual and reproductive health services?

Answered: 84 Skipped: 16

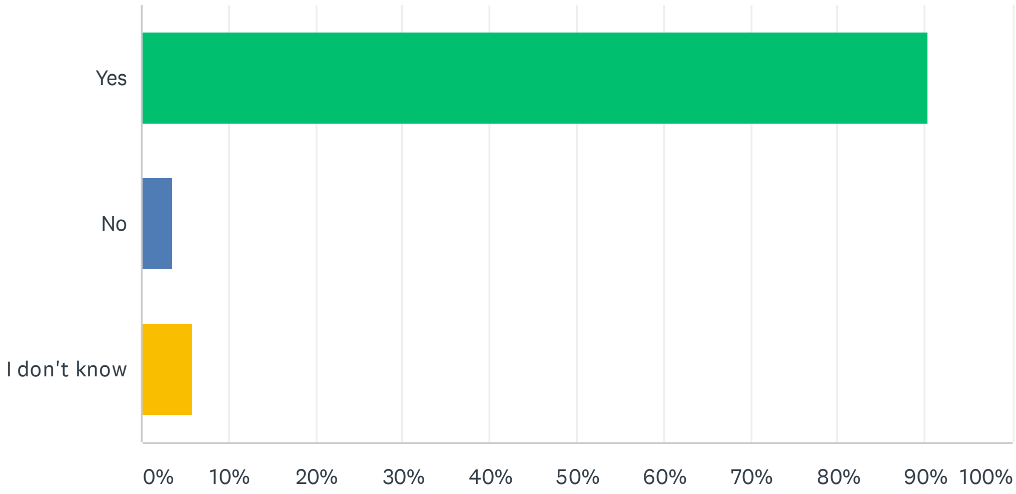

ANSWER CHOICES	RESPONSES	
Yes	90.48%	76
No	3.57%	3
I don't know	5.95%	5
TOTAL		84

Q24 Do you refer clients to a clinic for reproductive health services, including contraception and pre-conception care?

Answered: 84 Skipped: 16

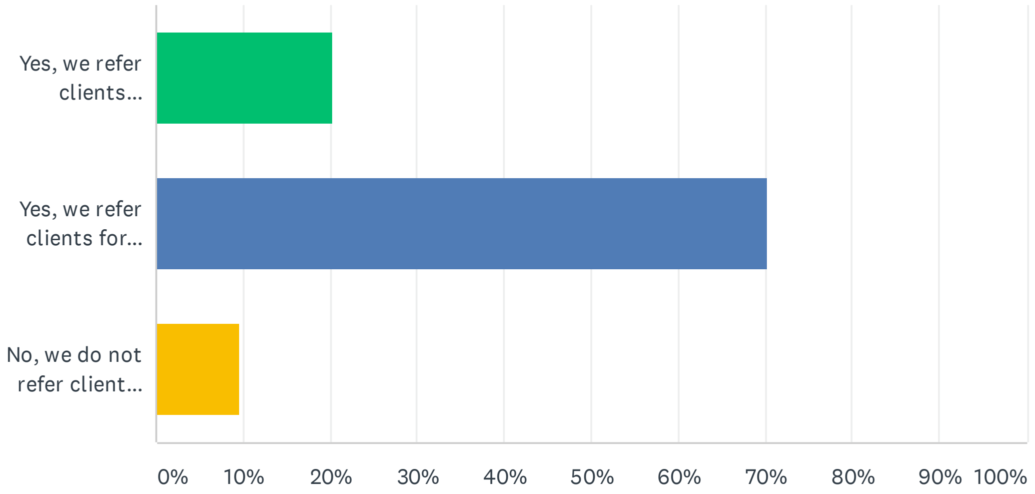

ANSWER CHOICES	RESPONSES	
Yes, we refer clients specifically for reproductive healthcare	20.24%	17
Yes, we refer clients for primary care, and intend that they receive reproductive healthcare from their primary provider, if needed	70.24%	59
No, we do not refer clients to clinics	9.52%	8
TOTAL		84

Q25 Do you provide any additional assistance to clients who wish to obtain reproductive health services? (Check all that apply)

Answered: 79 Skipped: 21

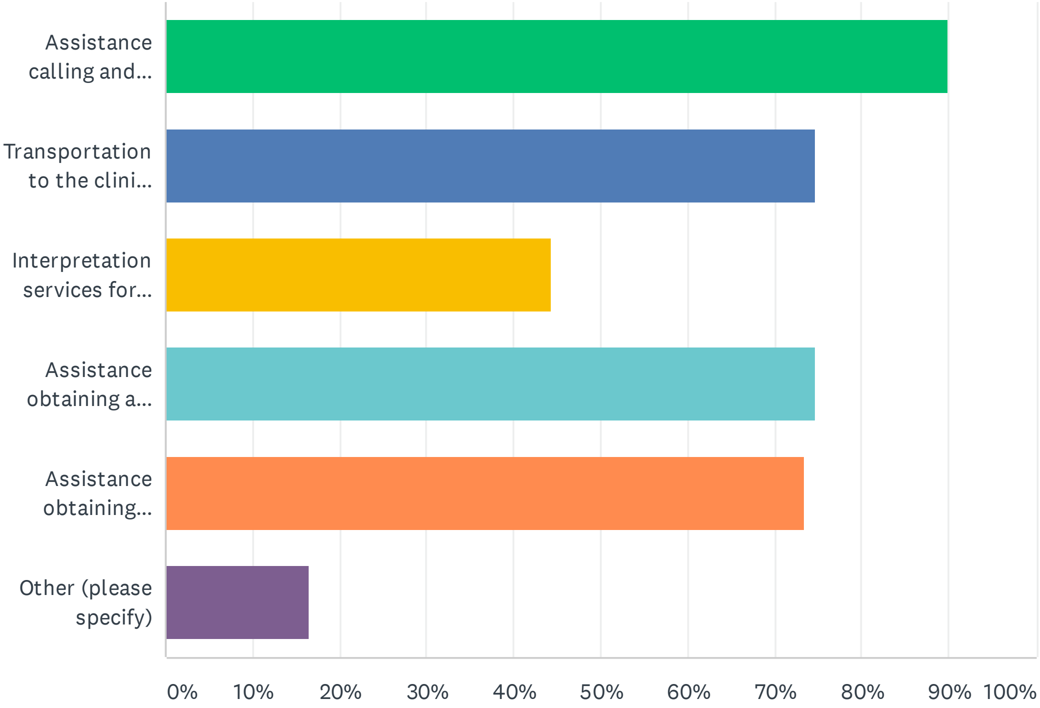

ANSWER CHOICES	RESPONSES	
Assistance calling and making an appointment	89.87%	71
Transportation to the clinic for appointments	74.68%	59
Interpretation services for clinical appointments	44.30%	35
Assistance obtaining a medication or filling a prescription	74.68%	59
Assistance obtaining health insurance or paying for healthcare services	73.42%	58
Other (please specify)	16.46%	13
Total Respondents: 79		

Q26 In your opinion, what are the barriers your office faces in ensuring women clients receive sexual and reproductive health information and services? (Check all that apply)

Answered: 80 Skipped: 20

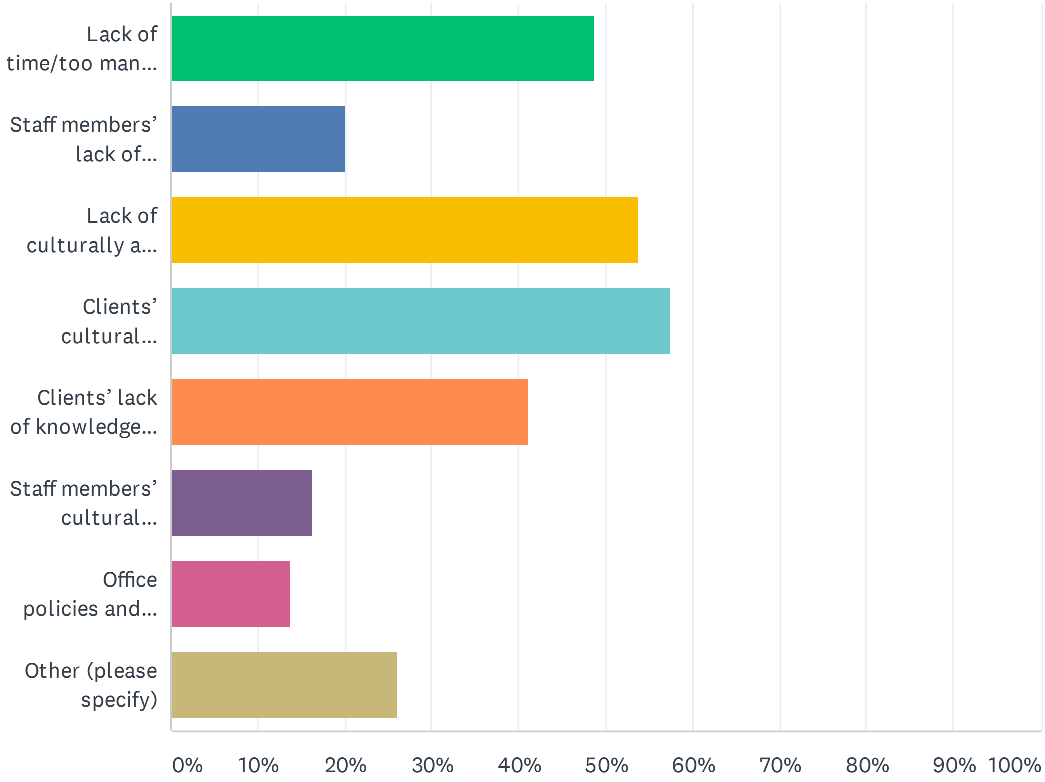

ANSWER CHOICES	RESPONSES	
Lack of time/too many competing priorities	48.75%	39
Staff members' lack of knowledge or skills discussing reproductive health	20.00%	16
Lack of culturally and linguistically appropriate materials about reproductive health to provide to clients	53.75%	43
Clients' cultural backgrounds, attitudes, and beliefs about reproductive health	57.50%	46
Clients' lack of knowledge about reproductive health	41.25%	33
Staff members' cultural backgrounds, attitudes, and beliefs about reproductive health	16.25%	13
Office policies and culture discourage discussing reproductive health topics	13.75%	11
Other (please specify)	26.25%	21
Total Respondents: 80		

Q27 In your opinion, do you think that your agency office would be interested in increasing its capacity to provide sexual and reproductive health information and referrals to clients?

Answered: 82   Skipped: 18

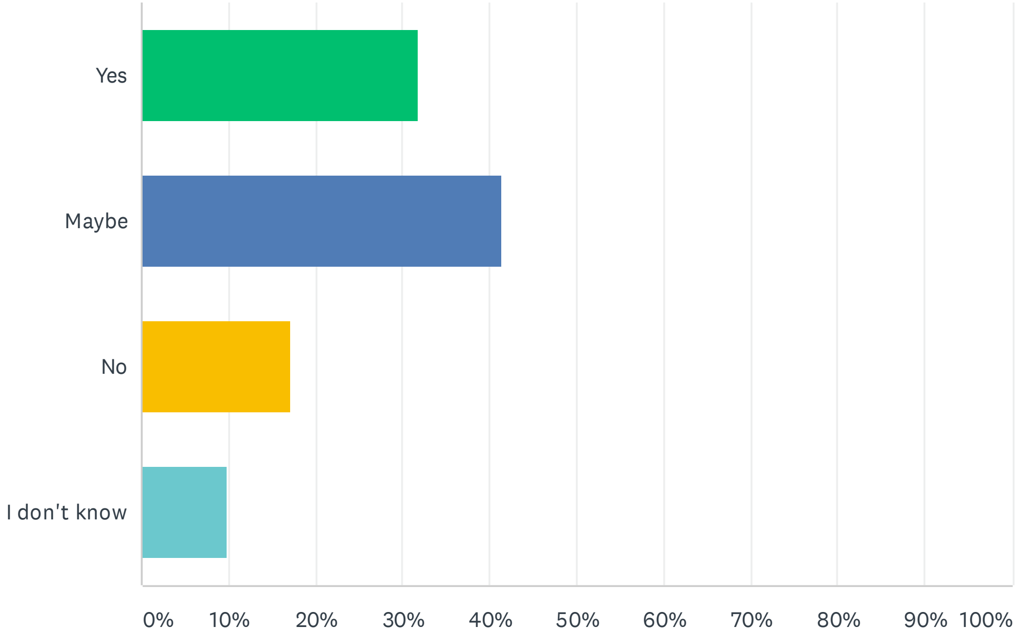

ANSWER CHOICES	RESPONSES	
Yes	31.71%	26
Maybe	41.46%	34
No	17.07%	14
I don't know	9.76%	8
TOTAL		82

Q28 What resources do you think would be most helpful to your agency office to increase your capacity to provide sexual and reproductive health information and referrals to clients? (Check all that apply)

Answered: 74 Skipped: 26

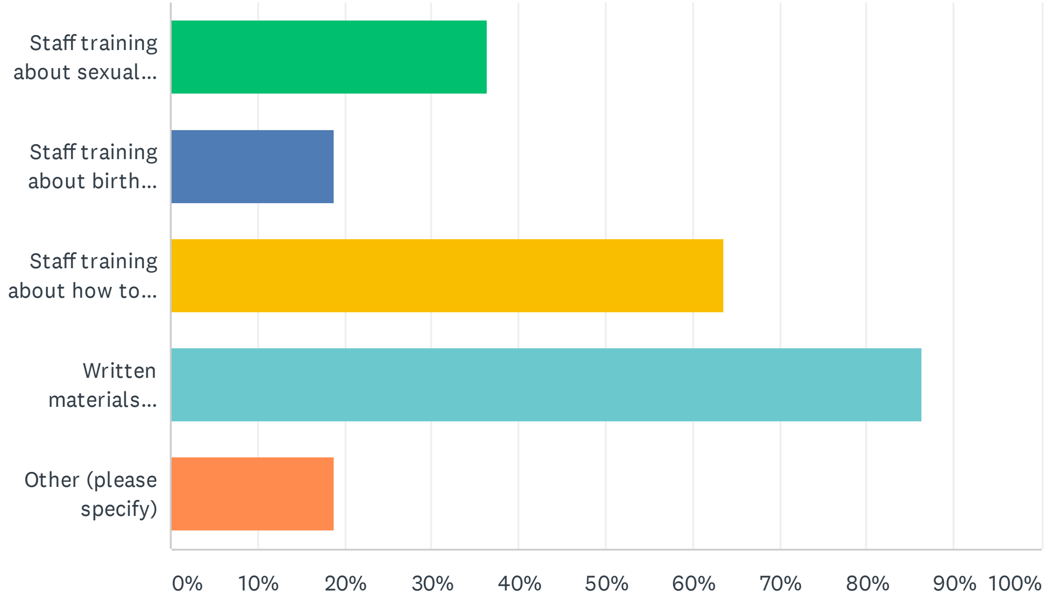

ANSWER CHOICES	RESPONSES	
Staff training about sexual and reproductive healthcare	36.49%	27
Staff training about birth control	18.92%	14
Staff training about how to talk to clients about sexual and reproductive health	63.51%	47
Written materials (handouts, pamphlets) for clients with sexual health information	86.49%	64
Other (please specify)	18.92%	14
Total Respondents: 74		

Q29 Contact information:

Answered: 71 Skipped: 29

ANSWER CHOICES	RESPONSES	
Name	97.18%	69
Company	67.61%	48
Address	69.01%	49
Address 2	8.45%	6
City/Town	69.01%	49
State/Province	70.42%	50
ZIP/Postal Code	67.61%	48
Country	0.00%	0
Email Address	69.01%	49
Phone Number	64.79%	46
