# Supplementary material for: Sexual and reproductive health information and referrals for resettled refugee women: A survey of resettlement agencies in the United States
Source: PLoS Med. 2021 May 3;18(5):e1003579. doi: 10.1371/journal.pmed.1003579 (PMC8092785; doi:10.1371/journal.pmed.1003579)
Supplement: S1 Checklist — (PDF) [file pmed.1003579.s003.pdf]

Standards for Reporting Qualitative Data Checklist

Checklist derived from: O'Brien B, Harris I, Beckman T, Reed D, Cook D. Standards for Reporting Qualitative Research. Academic Medicine. 2014;89(9),1245-1251.

**Title and Abstract**	
S1. Title	Sexual and reproductive health information and referrals for resettled refugee women: A survey of resettlement agencies in the United States
S2. Abstract	**BACKGROUND:** Refugee resettlement offices are the first point of contact for newly arrived refugees and play a significant role in helping refugees acclimate and settle into life in the United States. Available literature suggests that refugee women are vulnerable to poor sexual and reproductive health (SRH) outcomes, including STI and HIV infections as well as adverse pregnancy outcomes, but little is known about the role that refugee resettlement offices play in supporting refugee women's SRH. This study examines the capacity and interest of resettlement offices in providing SRH information and referrals to newly arrived refugees. **METHODS AND FINDINGS:** The research team conducted an online survey of staff members at refugee resettlement offices throughout the United States in 2018 to determine 1) available SRH resources and workshops; 2) referrals to and assistance with making appointments for SRH and primary care appointments; 3) barriers to addressing SRH needs of clients; and 4) interest in building the capacity of office staff to address SRH issues. The survey was created for this study and had not been previously used or validated. Survey data underwent descriptive analysis. A total of 236 resettlement offices were contacted, with responses from 100 offices, for a total response rate of 42%. Fifteen percent of refugee resettlement agencies who responded to the survey provide materials about SRH to clients, and 49% incorporate sexual health into the classes they provide to newly arrived refugee clients. Moreover, 12% of responding refugee resettlement agencies screen clients for pregnancy intention and 20% directly refer to contraceptive care and services. This study is limited by the response rate of the survey; 100 of 236 offices responded to the survey, but no conclusions can be drawn about those offices that did not respond. In addition, the survey instrument was not validated against any other sources of information about the practices of refugee resettlement offices. **CONCLUSIONS:** In this study, we observed that many resettlement offices do not routinely provide information or referrals for sexual and reproductive health needs.
**Introduction**	
S3. Problem formulation	Conflict and crisis have dire consequences on women and girls' sexual and reproductive health and rights. Women and girls affected by conflict often have limited access to sexual and reproductive health (SRH) care and are particularly vulnerable to unintended pregnancies, which may lead to high rates of

	unsafe abortion, maternal mortality, stillbirth, and perinatal mortality [citations in full article]. While migration to the U.S. may offer enhanced opportunities for access to SRH services, little is known about the extent of information and clinical services available for women through refugee resettlement agencies (RRAs). A study from 2004 showed that 25% of newly resettled refugee women were pregnant or had a reproductive health problem, and many had missed routine preventive care including breast and cervical cancer screenings [citations in full article]. Another study, published in Canada, calculated that 26% of newly resettled refugee women had unmet contraceptive need, meaning they were sexually active and not wanting to become pregnant but not using a contraceptive method [citations in full article]. These studies underscore that newly resettled refugee women have significant SRH needs. However, the authors were unable to find any previously published data about the role that RRA's play in supporting refugee women's SRH needs. *Introduction, paragraph 1.*
S4. Purpose or research question	To what degree are refugee resettlement offices providing sexual and reproductive health screening, education, and referrals to newly arrived refugee women? What are the barriers refugee resettlement offices face in providing these services to clients? How interested are refugee resettlement offices in increasing their capacity to provide sexual and reproductive health screening, education, and referrals? *Methods, paragraph 1.*
**Methods**	
S5. Qualitative approach and research paradigm	This study is a survey of the capacity of refugee resettlement offices for providing sexual and reproductive health screening, education, and referrals to newly arrived refugee women. Survey questions elicited qualitative data regarding the practices and procedures of refugee resettlement offices. *Methods, paragraph 1.*
S6. Researcher characteristics and reflexivity	Researchers only interacted with respondents by sending emails requesting participation with the survey. Researcher characteristics were not thought to influence results.
S7. Context	Refugee resettlement offices throughout the United States of America. *Methods, paragraph 2.*
S8. Sampling strategy	All refugee resettlement offices throughout the United States of America were asked to complete the survey; thus, no sampling strategy was employed for this study. *Methods, paragraph 2.*
S9. Ethical issues pertaining to human subjects	This study conducted a survey among adult respondents which gathered data solely regarding the practices and procedures at the respondents' place of employment. No personal or health-related data was collected. Thus, it was deemed that this study did not qualify as human subjects research and no ethics review was conducted. *Methods, paragraph 5.*
S10. Data collection methods	Data were collected through an online survey administered through the SurveyMonkey platform. *Methods, paragraphs 2 and 3.*

S11. Data collection instruments and technologies	Data were collected through an online survey administered through the SurveyMonkey platform. *Methods, paragraphs 2 and 3.*
S12. Units of study	As only one employee from each refugee resettlement office was requested to complete the survey, the unit of study is the refugee resettlement office itself. *Methods, paragraph 2.*
S13. Data processing	Data was processed using the descriptive analysis tools available on the SurveyMonkey platform. *Methods, paragraph 1.*
S14. Data analysis	Data was analyzed by descriptive analysis. *Methods, paragraph 1.*
S15. Techniques to enhance trustworthiness	Not applicable.
**Results/Findings**	
S16. Synthesis and interpretation	Across all of the 236 non-IRC and IRC offices invited to participate, the survey collected 100 responses, for a response rate of 42%. The response rate among the 210 non-IRC offices was 38%, while the response rate from the 26 IRC offices was 77%. Some respondents answered some but not all of the questions on the survey; for any given question, the response rate was between 80% and 96%. All nine of the resettlement agencies were represented among respondents, with IRC and the U.S. Conference of Catholic Bishops affiliates accounting for the largest percentage of respondents (Table 1). Survey respondents included resettlement managers, coordinators and directors, executive directors, deputy directors or vice presidents, program managers, and case managers. No demographic information about respondents was collected, aside from professional title/role. Seventy one percent of respondents provided the name of their office and contact information. Among those that did provide contact information, there were no known instances in which two people from the same office responded to the survey.<br>*Available SRH Resources or Workshops*<br>All survey respondents were asked whether their offices offered written materials (such as pamphlets or handouts) on SRH for clients. Of the 95 offices who responded to this question, 15% stated that their offices do have written materials, such as pamphlets or flyers (Figure 1). Of those offices that do have written SRH materials for clients, 92% reported providing materials in at least one language other than English. Only 5% of offices display any posters or signs that provide SRH information. Pamphlets and signs contained information on family planning/birth control and how to access clinical reproductive health services.<br>Forty-one percent of offices offer workshops that cover SRH topics. Among those offices, 51% offer workshops in a single session and 11% offer a multi-workshop SRH series. Forty-nine percent also include SRH content in cultural orientation courses. Forty percent of offices reported conducting classes at least four to six times per year. The most common topics covered include: family planning/birth control; the reproductive health system

	and how pregnancy occurs; and how to access clinical reproductive health services. Sixty-nine percent of workshops are always or usually separated by gender and 84% are voluntary for clients.<br>Only 17% of 86 responding offices have a health educator on staff. Only 12% of agencies surveyed always or usually screen female clients upon intake for whether they want to become pregnant in the next year.<br>Among the offices that responded, 52% reported developing partnerships with other organizations to provide SRH workshops.<br>*Referrals for SRH and Primary Care*<br>Almost all offices (90%) reported being aware of nearby clinics where clients can get SRH services, but only 20% reported that they refer clients to a clinic specifically for reproductive health services, including contraception and pre-conception care. However, when asked if the offices provided any additional assistance to clients who wished to obtain reproductive health services, 74% of respondents reported providing assistance with making appointments; transportation to clinics; assistance obtaining medications; and assistance obtaining health insurance. Forty-four percent of offices reported assisting their clients with having interpretation services during a clinical visit.<br>*Barriers to Addressing SRH needs*<br>Survey respondents were asked, "In your opinion, what are the barriers your office faces in ensuring women clients receive SRH information and services?" Primary responses included lack of time/too many competing priorities; lack of culturally and linguistically appropriate SRH materials; clients' cultural backgrounds, attitudes, and beliefs about SRH; and clients' lack of knowledge about reproductive health. Respondents cited materials and staff trainings as the most-needed resources in order to build their agencies' capacities to promote sexual health services for clients.<br>*Interest in Building Office Capacity*<br>Seventy-three percent of respondents reported either 'yes' or 'maybe' when asked if they thought their office would be interested in increasing its capacity to provide SRH information and referrals to clients (Figure 2). Those who responded positively were asked to select which listed trainings would be of potential interest to them; respondents could choose all that applied. Thirty-six percent selected general staff training about SRH, 19% selected training specifically about birth control, and 64% selected training about how to talk to clients about SRH. Additionally, 87% of respondents stated offices needed written SRH materials (handouts, pamphlets) for clients. *Results, paragraphs 1-8.*
S17. Links to empirical data	Not applicable.
**Discussion**	
S18. Integration with prior work,	The findings from this survey document that only a minority of resettlement offices are directly helping refugee women and

implications, transferability, and contribution(s) to the field	couples plan for their futures by planning their pregnancies and families or linking them to SRH services. For example, only 15% of responding agencies provide written SRH information to clients, and only 5% had posters or signs providing this information. Only 41% of offices provide SRH content in classes or workshops and just slightly more than 25% provide information specifically about family planning and contraceptive methods. While the majority of agencies refer clients for primary care and intend that refugees seek reproductive healthcare through that resource, only a small percentage of agencies refer clients to clinical resources specifically for reproductive healthcare. These findings highlight an oversight in the provision of care for refugee women, often times leaving the burden of seeking to understand, request and access SRH services solely to women themselves. This data points to a gap in services that are essential to women's ability to set and reach their goals in a new community, such as educational, career, and wellbeing goals. *Discussion, paragraphs 1-4.*
S19. Limitations	For this study, IRC affiliate offices were recruited through IRC's national headquarters, whereas non-IRC offices were recruited without the participation of their sponsoring agency, which likely explains the differential response rate between non-IRC and IRC resettlement offices. The high response rate among IRC offices makes it likely that the survey results accurately reflect the practices and resources available at all IRC offices. The same cannot necessarily be said of the non-IRC offices; with the lower response rate from non-IRC offices, study authors cannot definitively assert that the responses are representative of all resettlement offices. Also, the survey instrument was not validated against other sources of information regarding the practices of refugee resettlement offices. Additionally, while the survey did assess which topics were covered in written materials and posters, too few respondents answered these questions to allow for any meaningful discussion of material content. Finally, this study examines the provision of SRH information and education from the perspective of service providers, and not the experiences of refugee women themselves. *Discussion, paragraph 5.*
**Other**	
S20. Conflicts of interest	The researchers report no conflicts of interest in conducting this study.
S21. Funding	This study was funded by Advocates for Youth.
